# Transmission phenotype of *Mycobacterium tuberculosis* strains is mechanistically linked to induction of distinct pulmonary pathology

**DOI:** 10.1371/journal.ppat.1007613

**Published:** 2019-03-06

**Authors:** Sheetal Verma, Kamlesh Bhatt, Arianne Lovey, Rodrigo Ribeiro-Rodrigues, Joan Durbin, Edward C. Jones-López, Moises Palaci, Solange A. Vinhas, David Alland, Reynaldo Dietze, Jerrold J. Ellner, Padmini Salgame

**Affiliations:** 1 Rutgers University-New Jersey Medical School, Department of Medicine, Centre for Emerging Pathogens, Newark, New Jersey, United States of America; 2 Cellular and Molecular Immunology Laboratory, Núcleo de Doenças Infecciosas, Universidade Federal do Espírito Santo, Vitória, Brazil; 3 Rutgers University-New Jersey Medical School, Department of Pathology, Newark, New Jersey, United States of America; 4 Section of Infectious Diseases, Department of Medicine, Boston Medical Center and Boston University School of Medicine, Boston, Massachusetts, United States of America; 5 Mycobacteriology Laboratory, Núcleo de Doenças de Infecciosas, Universidade Federal do Espírito Santo, Vitória, Brazil; 6 Núcleo de Doenças Infecciosas, Universidade Federal do Espírito Santo, Vitória, Brazil; 7 Global Health & Tropical Medicine, Instituto de Higiene e Medicina Tropical, Universidade Nova de Lisboa, Lisbon, Portugal; McGill UniversityHealth Centre, CANADA

## Abstract

In a study of household contacts (HHC), households were categorized into High (HT) and Low (LT) transmission groups based on the proportion of HHC with a positive tuberculin skin test. The *Mycobacterium tuberculosis* (Mtb) strains from HT and LT index cases of the households were designated Mtb-HT and Mtb-LT, respectively. We found that C3HeB/FeJ mice infected with Mtb-LT strains exhibited significantly higher bacterial burden compared to Mtb-HT strains and also developed diffused inflammatory lung pathology. In stark contrast, a significant number of mice infected with Mtb-HT strains developed caseating granulomas, a lesion type with high potential to cavitate. None of the Mtb-HT infected animals developed diffused inflammatory lung pathology. A link was observed between increased *in vitro* replication of Mtb-LT strains and their ability to induce significantly high lipid droplet formation in macrophages. These results support that distinct early interactions of Mtb-HT and Mtb-LT strains with macrophages and subsequent differential trajectories in pathological disease may be the mechanism underlying their transmission potential.

## Author summary

*Mycobacterium tuberculosis* (Mtb), the bacteria that causes tuberculosis (TB), is spread through the air from infected patients to their close contacts. In a household contact (HHC) study of patients with TB, we found that in some households a larger proportion of contacts were infected with Mtb compared to other households. We categorized the households into High (HT) and Low (LT) transmission groups. The Mtb strains obtained from the TB patients of the HT and LT households were designated Mtb-HT and Mtb-LT, respectively. In this study, we investigated in a mouse model of TB, the mechanistic basis for the variability in transmission of Mtb from infected patients to their close contacts. We found that C3HeB/FeJ mice infected with Mtb-LT strains developed diffused inflammatory lesions characteristic of granulocytic tuberculous pneumonia. In stark contrast, a significant number of mice infected with Mtb-HT strains developed caseating granulomas, a lesion type with high potential to cavitate. In conclusion, we report that the segregation of Mtb strains into high and low transmission phenotype is mechanistically linked to their ability to induce distinct pulmonary pathology.

## Introduction

Mtb is one of the most successful pathogens known; yet mechanisms underlying variability in transmission remain poorly understood. Pioneering work from Riley and colleagues [1] using TST conversion in guinea pigs that were exposed to air from a TB ward as a measure of infectiousness, found significant variability in infectiousness among untreated patients with drug susceptible Mtb. Despite having comparable sputum positivity, only 8 of the 61 patients in the TB ward were found to transmit infection [1]. Using a similar model of air sampling analysis in test animals, a great degree of variability in infectiousness was also reported for HIV-infected patients with drug susceptible and resistant TB [2–4]. Subsequent studies demonstrated that some patients transmit their infection to large numbers of contacts whereas other patients transmit rarely or not at all (even after controlling for factors such as extent of disease in the index case and length of exposure) [5–10]. Variability in transmission could result from differences in the variability of infectious aerosols produced during coughing by patients with pulmonary tuberculosis [11].

*Mycobacterium tuberculosis* Complex (MTBC) comprises of six human adapted lineages and a recently discovered lineage 7 [12]. Whole genome sequence data show that the lineages differ in their content of SNPs, small insertion and deletions, large genomic deletions, large duplications and insertion sequences [12]. Several studies have addressed whether Mtb genotypic diversity is associated with diversity in clinical outcome [reviewed in [12, 13]]. Overall, lineage 2 was found to be highly associated with virulence and transmission in different ethnic populations [14–20]. However, other studies did not find a higher fitness for Beijing strains of lineage 2 [21–23]. For example, in a cohort study of patients with TB and their HHC in The Gambia where *M*. *africanum* is endemic, there was no difference in transmission between *M*. *africanum* and Mtb or between the MTBC lineages [24]. In another population-based study in Montreal, Canada, four Mtb lineages were identified- Euro-American (lineage 4), Beijing (lineage 2), Indo-Oceanic (lineage 1) and East African-Indian (EAI-lineage 3) [25]. In contrast to previous studies, higher transmissibility of the Beijing lineage was not observed in this study. However, the EAI lineage was associated with lower rates of TB transmission, as measured by positive TST among close contacts of pulmonary TB cases [25]. The Beijing genotype consists of a number of sub-lineages and so the discrepant findings could be due to the prevalence of different sub-lineages in the different study populations, as suggested previously [12]. For example, in a study conducted in Western Cape, South Africa, significantly higher transmission was found to be linked to recently evolved sub-lineages of the Beijing strain family than to other sub-lineages, indicating that strains within individual lineages have acquired distinct transmissibility traits [17]. Similarly, a study from China also concluded that transmissibility was dissimilar among the Beijing sub-lineages [26]. Consistent with the idea that there is heterogeneity within a lineage, studies in an animal model of transmission reported that Beijing genotype strains exhibited various degrees of virulence phenotype and transmissibility [26]. It has been suggested that the heterogeneic immune response and virulence of the Beijing strains may be due to their differential engagement of the innate Toll-like receptors (TLR) [27].

The high transmissibility and prevalence of some sub-lineages belonging to the Beijing phenotype raises the question of whether the type of immune response elicited by some strains in the lineage gives them a selective advantage over other genotypes. In this regard, strains within the highly prevalent “modern” Beijing genotype have been linked to low inflammatory cytokine responses [28–30] and faster growth rate *in vitro* [31, 32] compared to strains within the “ancient” Beijing genotype. By selecting representative strains from the Modern (lineage 2, lineage 3 and lineage 4) and ancient lineages (lineage 1, lineage 5 and lineage 6), Portevin et al. [30] extended the analysis of macrophage cytokine responses to all the major phylogenetic lineages. They found that overall, strains from the modern lineages induced lower levels of pro-inflammatory cytokines when compared with strains representing ancient lineages. Furthermore, two strains, HN878 (lineage 2), a member of the Beijing family that caused several outbreaks of TB in Texas [33, 34] and Mtb strain CH (lineage 3) that caused a large outbreak of TB in Leicester, UK [35] also exhibited a low inflammatory phenotype. In contrast, CDC1551 (lineage 4) that also caused a large number of TB infections in a small rural community in Tennessee [36], induced a robust inflammatory response and was less virulent in mice [37]. The finding with CDC1551, together with several studies showing lack of association of “modern” lineages with disease presentation [38–41] or transmission [21–24], indicates an incomplete understanding of bacterial factors that favor transmission success.

Many factors, including source infectiousness [42], cough aerosols [43] and nature and proximity of contact [44] affect transmission. To address pivotal questions pertaining to transmissibility of different Mtb strains, we conducted a study that included 731 household contacts (HHC) of 124 infectious TB patients, and found marked heterogeneity in Mtb transmission within households [42]. Index cases (with pulmonary TB disease) and their respective households were categorized into high (HT) and low (LT) transmission groups based on the proportion of HHC with a positive tuberculin skin test. The Mtb strains from HT and LT index cases of the households were designated Mtb-HT and Mtb-LT, respectively. The goal of this study was to explore the role of Mtb strain in the observed differences in transmission and the mechanistic basis by which the epidemiologically characterized Mtb-HT and Mtb-LT strains, though belonging to lineage 4, have diversified in their transmission profile. The C3HeB/FeJ mouse model was employed to examine the role of Mtb strain in the dichotomous transmission outcomes of the HT and LT households.

Although there are disparities in susceptibility to infection and disease manifestations that exist between humans and animals, experimental models such as C3HeB/FeJ mice that present with lung pathology more typical of human TB disease [45–49] are useful for hypothesis-driven research aimed at understanding TB immunopathogenesis. Using the C3HeB/FeJ mouse model, we found that Mtb-HT and Mtb-LT strains induced distinct growth pattern and pathological disease that fit their transmission phenotype. We also found increased lipid biogenesis in Mtb-LT infection of macrophages compared to Mtb-HT infection suggesting that this difference in host response may be a factor in the divergence in infection outcome between the two groups of animals.

## Results

### Household contact study

As previously reported, the HHC study only included crowded (≥3 HHC) dwellings with an intense (≥3 weeks of cough) and homogeneous (sputum AFB ≥2) infectious exposure, and classification of households as “high” or “low” transmission was based on TST positivity of contacts at the end of enrollment [42]. Briefly, the percentage of contacts from a given household that had a TST≥ 10mm of induration either at baseline or by 8–12 weeks was used to determine the transmission category of the household. If there were ≥70% TST positivity in HHCs, the active TB disease patient or “index case” was considered “High Transmission” (HT) and if there was ≤40% contacts that were TST positive, the index case was considered “Low Transmission” (LT) [42]. 293 TB patients were screened and 124 index cases were enrolled. The Mtb strain isolated from HT index case was designated Mtb-HT and from LT index case was designated Mtb-LT.

From the panel of HT and LT strains, three strains from each group were randomly picked, blinded to any patient or household contact details. Based on subsequent examination of the index case and household characteristics, it is reasonable to assume that the six randomly selected strains are representative of the larger groups of HT and LT strains. All of the HT and LT strains belong to lineage 4. The index case and household characteristics of the six strains are described in Table 1. Total TST positivity was 100% for the HT index cases and 14–40% for the LT cases (Table 1). All of the six isolates were collected from the Vitória Metropolitan area. Vitória, Brazil is the capital city of Espírito Santo, a state with a TB incidence rate of 38/100,000 and very low HIV prevalence in TB cases (<2%) and the general population (<1%). While migration into this region is limited, there is a high level of inter-municipality mobility amongst inhabitants. We previously showed that there was no relationship between the HT and LT transmission phenotypes and the presence of RFLP/spoligotyping clusters in the community [50]. As shown in Table 1, the six strains were unique isolates or belonged to different clusters. Based on RFLP, Mtb-HT1 and Mtb-LT1 are different even though they both fall within the LAM9 sub-lineage. This sub-lineage is the most common spoligotyping sub-lineage in Brazil. Based on radiographic appearance, HT1 and HT3 index cases had far advanced lung disease and HT2, LT1, LT2 and LT3 index cases had moderately advanced lung disease. Chest X-rays of the TB patients showed that HT1, HT2 and HT3 index cases had cavitations, whereas only patient LT1 presented with cavitary disease (Table 1). For *in vitro* assays, seven additional strains isolated from HT and LT index cases were tested (S1 Table).

**Table 1:** Index case and HHC data of Mtb strains selected for mouse infections.

	Mtb-HT1	Mtb-HT2	Mtb-HT3	Mtb-LT1	Mtb-LT2	Mtb-LT3
RFLP and Spoligotyping family of Mtb strains	Non cluster/LAM9	Cluster/LAM4	Non cluster/Orphan	Cluster/LAM9	Cluster/H3	Cluster/LAM5
**Index case data of the Mtb strain**						
Age	36	33	19	36	41	23
Gender	Male	Male	Female	Female	Male	Male
Sputum smear	3+	3+	3+	3+	2+	3+
Disease Extent, 1 = less advanced, 2 = moderately advanced, 3 = far advanced	3	2	3	2	2	2
Cavitation (as seen on CXR)	Present	Present	Present	Present	Absent	Absent
Presence of fibrosis	Yes	Yes	No	No	No	No
Pleural Thickening	Yes	No	No	No	Yes	Yes
**Data from HHC of the Index cases**						
Number of HHC	3	3	9	5	7	5
1^st^ TST positive (n,%)	3/3 (100)	3/3 (100)	8/8 (100)*	1/5 (20)	1/7 (14)	1/5 (20)
2^nd^ TST positive (n,%)				2/5 (40)	1/7 (14)	1/5 (20)
IGRA positive (n,%)	2/3 (67)	3/3 (100)	6/8 (75)*	0/5 (0)	0/5 (0)^‡^	1/4 (25)^#^

* One HHC missing TST and IGRA results

^#^ One HHC missing IGRA result

^‡^ Two HHC missing IGRA result

### Mice infected with Mtb-HT and Mtb-LT strains show differential pattern of bacterial growth

**Fig 1:**
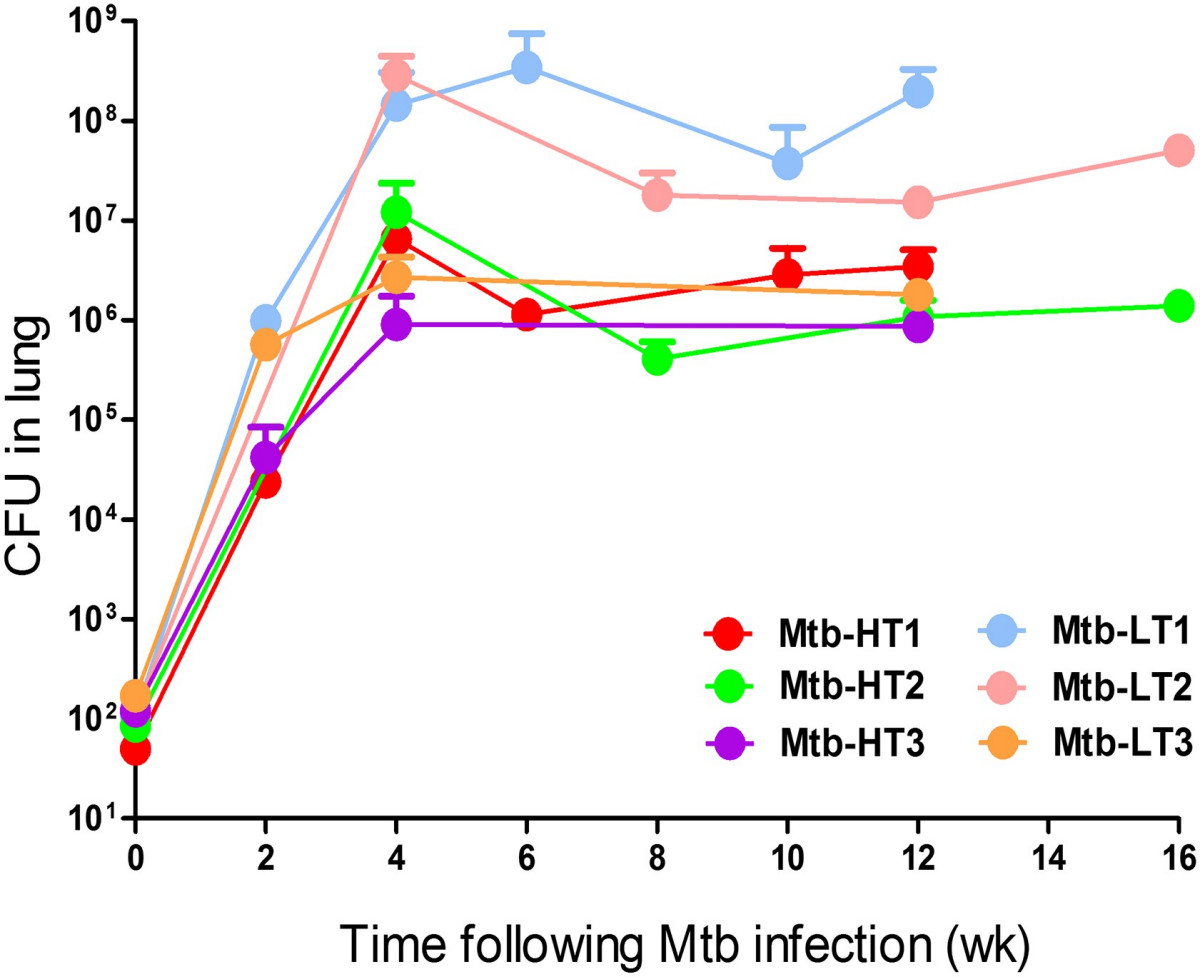
Comparison of bacterial burden in the lungs and peripheral organs of C3HeB/FeJ mice infected with Mtb-HT and Mtb-LT strains. Mice were infected with a low dose inoculum (50–120 CFU) with the three Mtb-HT and three Mtb-LT strains. 5 mice were included for each time point. At indicated time points following aerosol infection, lungs were harvested, and serial dilutions of lung homogenates were plated on 7H11 agar plates and the bacterial load was determined between 28–35 days of incubation at 37°C. Two-way ANOVA showed significant difference between animals infected with HT and LT groups. Week 2: LT-1 vs HT-1, HT-3 (p < 0.01); week 4: LT-2 vs HT-1, HT-2, HT-3, LT-3 (p < 0.0001); week 6: LT-2 vs HT-1, HT-2, HT-3, LT-3 (p < 0.0001); week 12: LT-1 vs HT-1, HT-2, HT-3, LT-3 (p < 0.05).

In separate infections, previously grown stocks of the six Mtb clinical strains described in Table 1 were used to infect C3HeB/FeJ mice. Bacterial burden was monitored at different time-points following aerosol infection of the mice (Fig 1). We observed that despite similar inoculum size, lung bacterial burden in mice infected with two of the three Mtb-LT strains, Mtb-LT1 and Mtb-LT2, were significantly higher than all three HT strains at all time points sampled (Fig 1). Mtb-LT3 produced higher bacterial growth at week 2 post infection but was not significantly different from Mtb-HT strains at later time points (Fig 1). These changes in bacterial growth were also reflected in extra-pulmonary dissemination to the spleen and liver (S1 Fig). A repeat experiment with Mtb-HT1 and Mtb-LT1 also showed that CFU in the lungs, mediastinal lymph nodes and spleen at 12 weeks of infection was significantly higher in Mtb-LT1 infected mice compared with Mtb-HT1 infected mice (S2 Fig).

To delineate whether host genotype contributed to the differences between Mtb-HT and Mtb-LT strains, we also infected inbred C57BL/6 and BALB/c mice with Mtb-HT1 and Mtb-LT1 strains. In both genotypes of mice, we observed that compared to Mtb-HT1, Mtb-LT1 infected C57BL/6 and BALB/c mice showed significantly higher CFU in the lungs (S3A Fig).

### Infection with Mtb-LT strains generates a strong inflammatory response in the lungs

**Fig 2:**
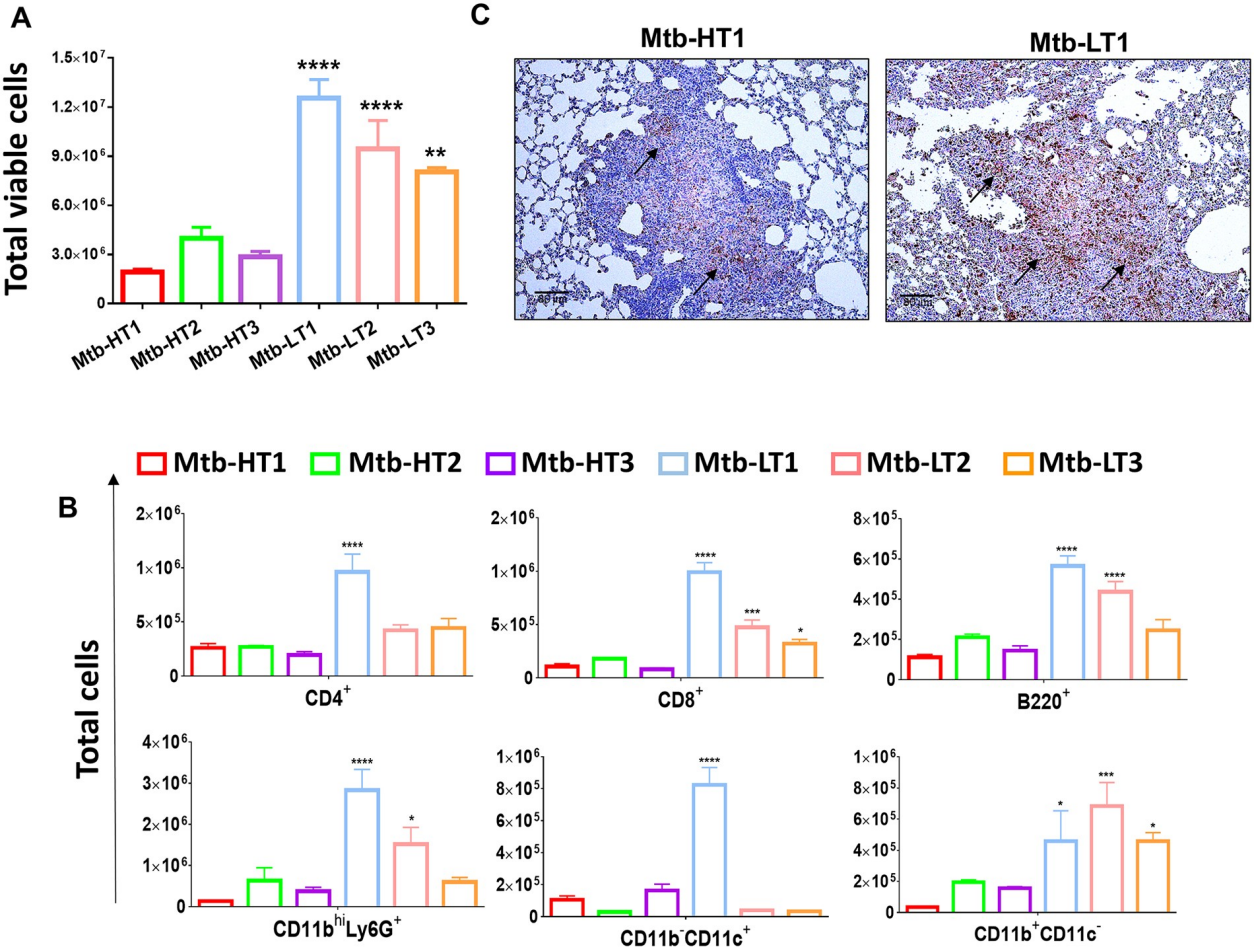
Increased inflammatory response and cellular infiltration in Mtb-LT infected mice. Single cell preparations were obtained, and trypan blue exclusion method was used to calculate the total live cells in the lungs of Mtb-HT and Mtb-LT infected C3HeB/FeJ mice at 4 weeks post-infection (A). Lung cells from 4 week-infected mice were surface stained with directly conjugated antibodies to evaluate the total number of immune cell subsets by flow cytometry (B). Immunostaining with anti-Ly6G performed on paraffin-embedded fixed lung tissue sections from Mtb-HT and Mtb-LT infected mice. The image is a representative of three animals from each of the six infections. (C). For A and B, data are from five mice and presented as mean +/- SEM. One-way Anova was used to compare HT-1 infection with the rest of the groups.

Next, we evaluated the composition of the cellular infiltrates to the lungs of 4 week-infected C3HeB/FeJ mice. We observed that all three strains of Mtb-LT infected C3HeB/FeJ mice had significantly higher number of total viable cells in the lungs as compared to the mice infected with the three Mtb-HT strains (Fig 2A). Further analysis of the cellular composition of the recruited cells showed that, compared to Mtb-HT1, all three Mtb-LT infected mice had significantly increased numbers of CD8^+^ T cells and CD11b^+^CD11c^+^ recruited macrophages while B220^+^ B-cells, CD11b^hi^Ly6G^+^ neutrophils were increased in Mtb-LT1 and Mtb-LT2 infections. CD4^+^ T cells and CD11b^-^CD11c^+^ alveolar macrophages were significantly enhanced only in Mtb-LT1 infection (Fig 2B). There was no significant increase in any cellular subsets in Mtb-HT2 and Mtb-HT3 infections compared to Mtb-HT1 (Fig 2B). The increase in neutrophil numbers in Mtb-LT1 infection was confirmed in immunohistochemical staining of lung sections. A large number of Ly6G^+^ neutrophils were observed in the granulomatous lesions of four week Mtb-LT1 infected mice compared to Mtb-HT1 infected mice that had sparse neutrophils in the granuloma (Fig 2C). Consistent with increased cellular recruitment, evaluation of lung homogenates showed an overall increase in TNF, IL-1β, IL-6, IL-17 and KC (CXCL1) in Mtb-LT infected mice at four weeks following infection compared with Mtb-HT infected mice (S4 Fig). These data indicate that Mtb-LT strains induce an overall strong inflammatory response, admittedly, though, this could be an indirect effect as a result of the increased bacterial burden in these mice.

### Development of distinct lung immunopathology between animals infected with Mtb-HT and Mtb-LT strains

**Fig 3:**
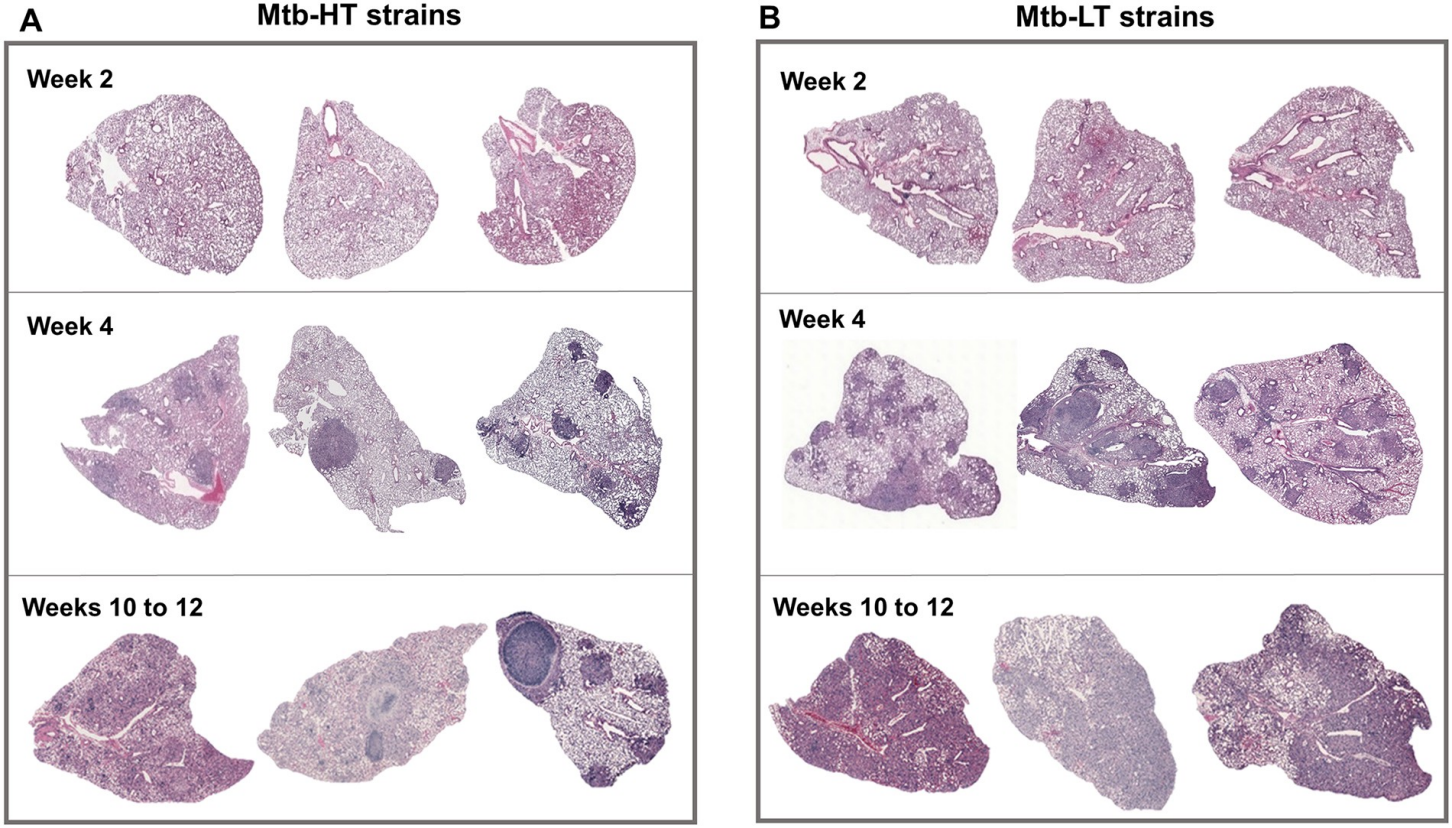
Differential granulomatous response in C3HeB/FeJ mice infected with Mtb-HT and Mtb-LT strains. Mosaic H&E images of lung tissue representing acute (2–4 weeks) and chronic (10–12 weeks) phases of infection from the three Mtb-HT infections (A) and three Mtb-LT infections (B). Mosaic images were created using Surveyor software with Turboscan by objective imaging at 20X. Each group includes 4–5 mice per time point. Each time point includes representative images from all three Mtb-HT and Mtb-LT infections.

H&E staining of paraffin-embedded lung sections revealed that there were striking differences in the types of granulomatous lung lesions that were formed between animals infected with Mtb-HT and Mtb-LT strains. Although early on, there were no clear differences in lung pathology between the Mtb-HT and Mtb-LT infected mice (Fig 3A and 3B, top panel), at four weeks post-infection Mtb-HT infected mice had well-defined, circumscribed lesions in otherwise normal parenchyma. In contrast, diffuse granulomatous inflammation was observed in Mtb-LT infected animals (Fig 3A and 3B, middle panel). This is consistent with the enhanced cytokine and chemokine induction, and neutrophil accumulation in Mtb-LT infected mice. During the chronic stages of infection (weeks 8–12), well-formed granulomas in the lungs of Mtb-HT infected mice were seen to expand, whereas Mtb-LT infected mice showed widespread tissue destruction (Fig 3A and 3B, bottom panel).

**Fig 4:**
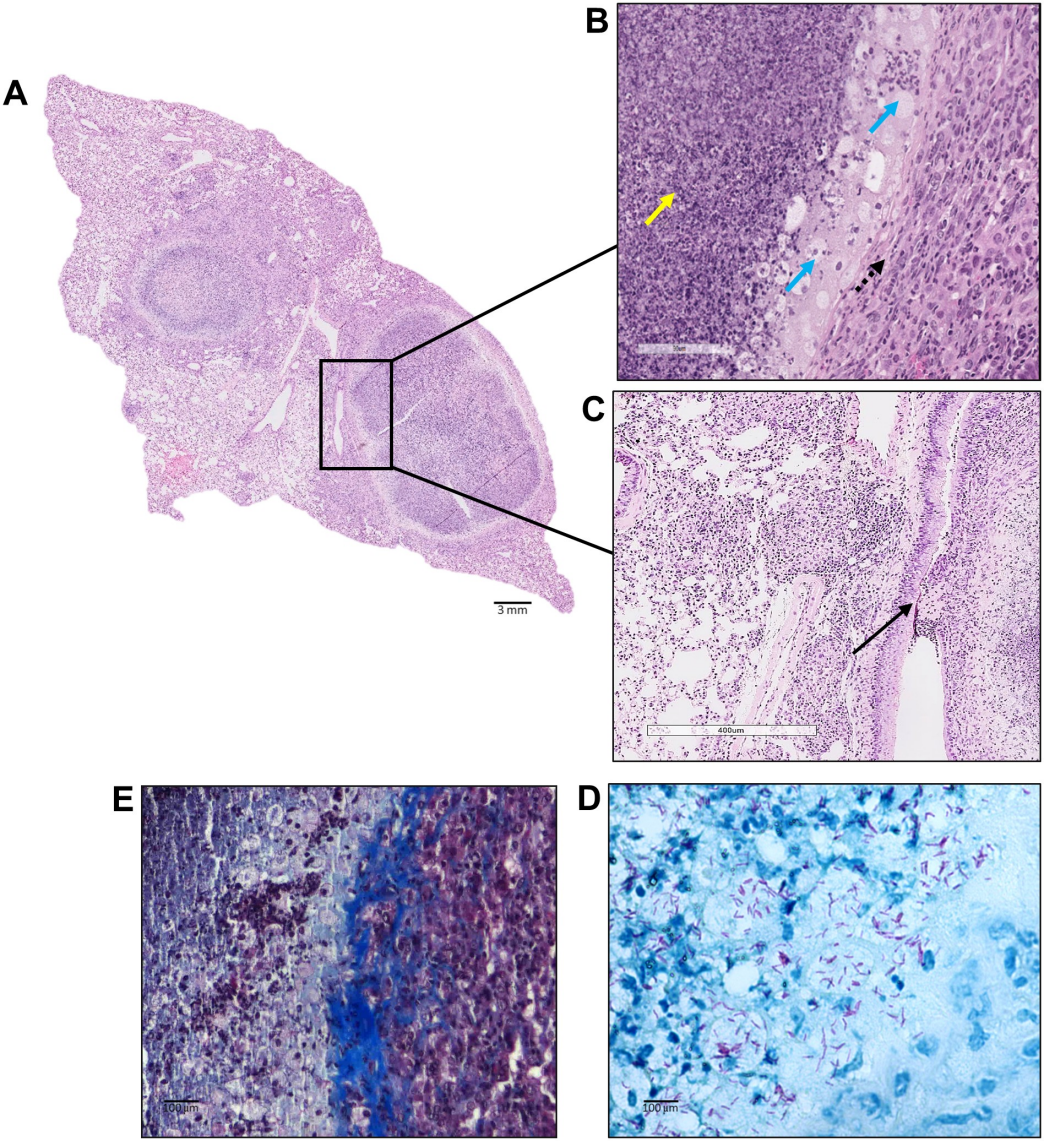
Mtb-HT infected C3HeB/FeJ mice form caseating granulomas. Representative histological image of murine lung infected with Mtb-HT1 during chronic infection, show distinctive fibrotic and necrotic lesions (A). The zoomed in area demonstrates the extent of inflammation observed in these granulomas. Presence of necrotic debris in the center core (yellow arrow), foamy macrophages (cyan arrow) and surrounding layer of fibroblasts (dashed arrow) and is seen in the granulomas (B). Solid arrow points to outer edge of granuloma fusing with an airway (C). Representative image of AFB staining at 12 weeks post infection (D). Serial sections were stained using Trichrome staining and show collagen deposition (seen in blue) on the outer periphery of the granuloma (E). Leica SCN400 F whole-slide scanner was used for scanning histological sections up to 40X magnification and images were analyzed using Aperio ImageScope.

**Fig 5:**
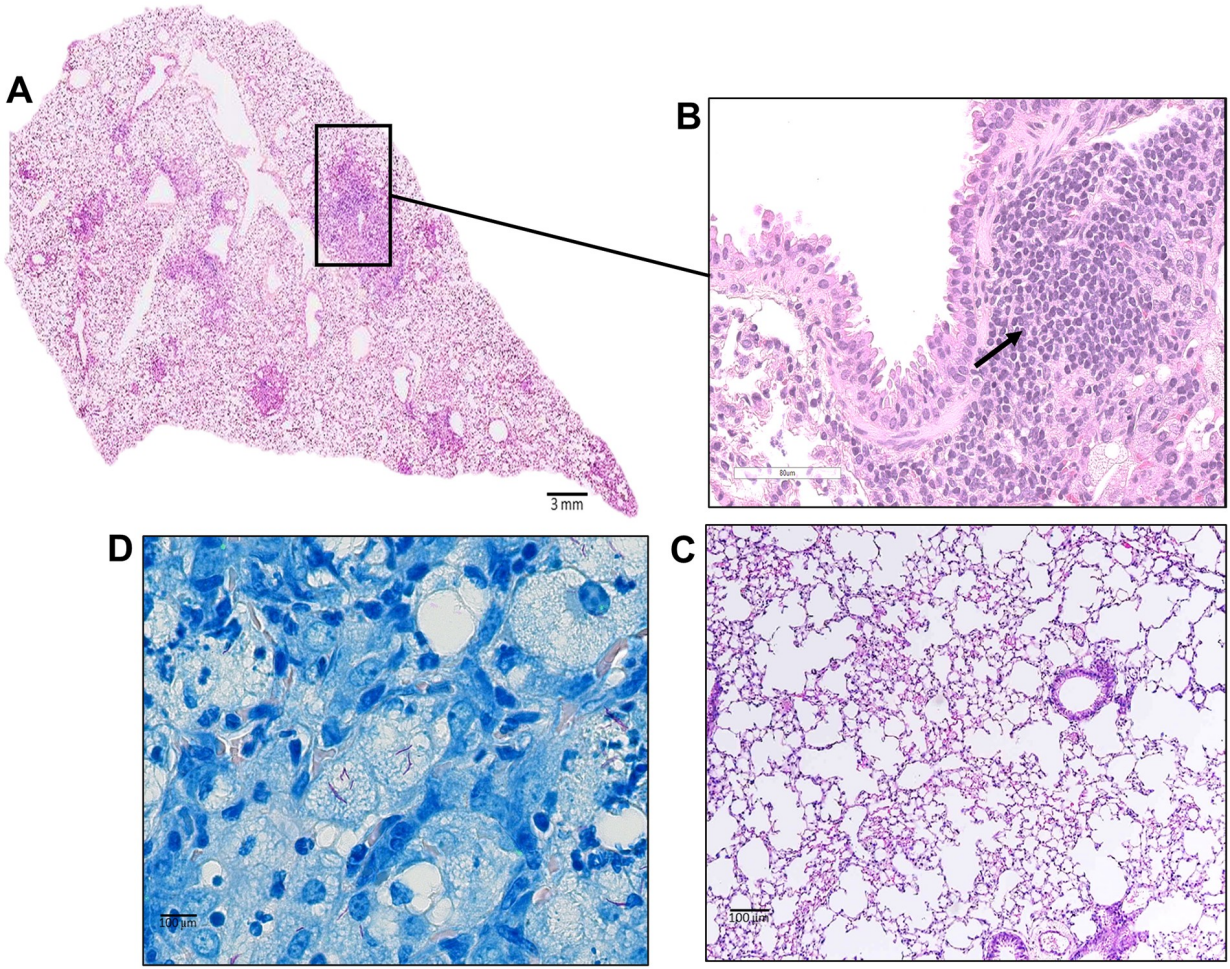
Mice infected with Mtb-HT strains show presence of solid discrete granulomas. H&E stained sections of formalin-fixed paraffin-embedded tissues from the lungs of Mtb-HT infected C3HeB/FeJ mice obtained at 12 weeks post infection show well defined tubercle granuloma (square box) (A). Higher magnifications zoomed-in images show presence of peribronchiolar lymphocytes (B) and open alveolar spaces with less inflamed alveolar septae (C). AFB staining (D). High magnification images were taken using a Leica DM6000B by objective imaging at 20X and 100X and also by analyzing high-magnification scanned images using Aperio ImageScope. These images are representative of pathology seen with all three Mtb-HT infections.

**Fig 6:**
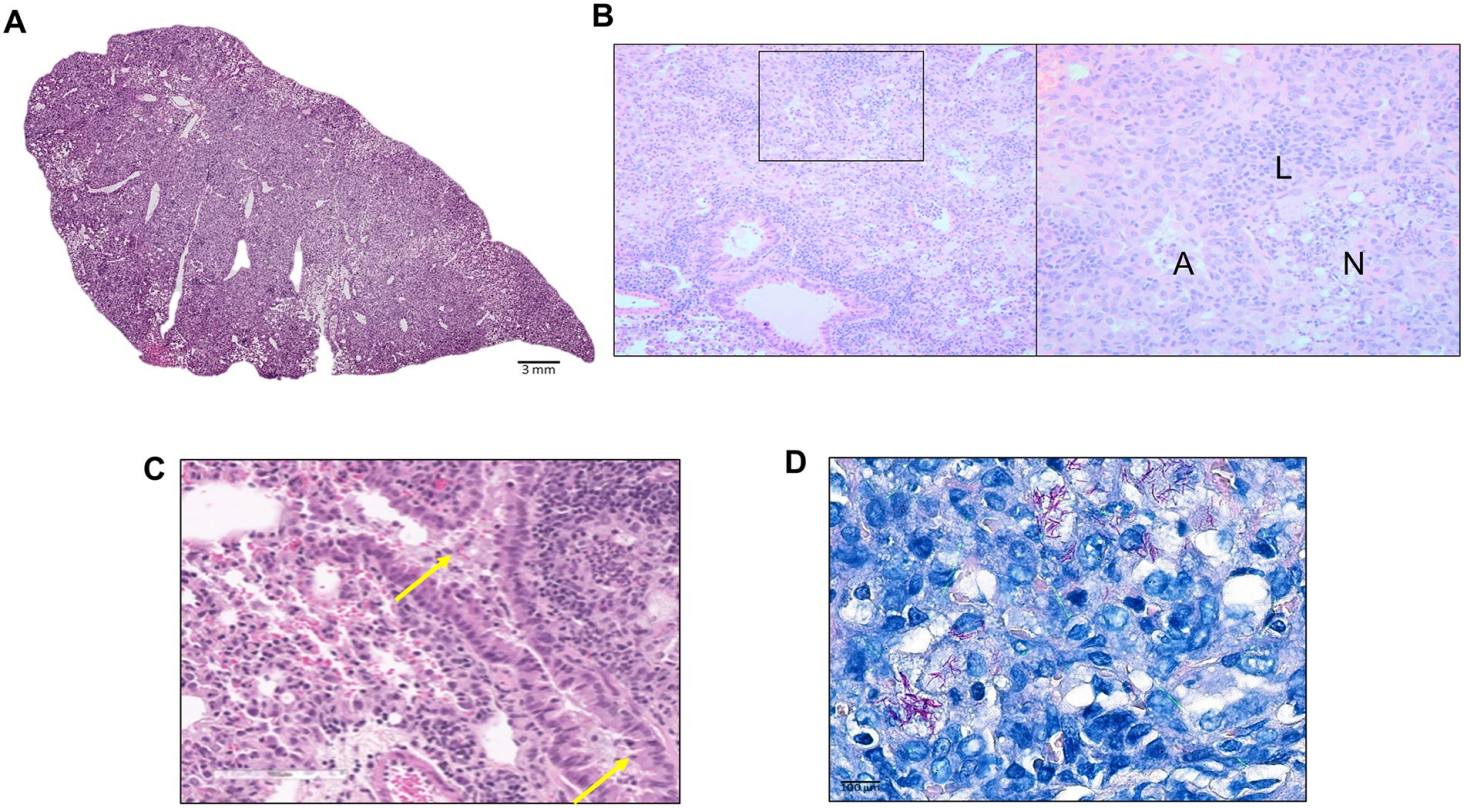
Infiltrative pathology in the lungs of Mtb-LT-infected C3HeB/FeJ. H&E stained sections of formalin-fixed paraffin-embedded tissues from the lung of Mtb-LT infected C3HeB/FeJ mice obtained at 12 weeks post infection (A). Zoomed in area showing necrotic areas with karyorrhectic debris with alveolar space “A”, lymphocytic cluster “L” and neutrophilic debris “N” (B), and cellular exudate in airways (C). High magnification images were taken using a Leica DM6000B by objective imaging at 20X and 100X also by analyzing high-magnification scanned images using Aperio ImageScope. Ziehl-Neelsen acid-fast staining (D). These images are representative of pathology seen with all three Mtb-LT infections.

At the latest time point, caseating granulomas in the HT-infected mice were well-defined, circumscribed lesions surrounded by a thin layer of fibroblasts and collagen, in otherwise normal parenchyma (Fig 4A). Many of these lesions were in close proximity to intact airways (Fig 4C), spilling bacterial and necrotic debris into the airway lumen. Surrounding the necrotic center were acid fast organisms (Fig 4D) and collagen-rich cell debris (Fig 4B and 4E) enriched with foamy macrophages within a collar of fibroblasts (Fig 4B). Many of the granulomas in Mtb-HT infected mice appeared as small aggregates of cells (Fig 5A), dominated by lymphocytes (Fig 5B), minimal inflammation (Fig 5C) and containing few intracellular bacteria (Fig 5D). In contrast to the discrete granulomas observed in mice infected with Mtb-HT strains, there was diffuse lung pathology in mice infected with the Mtb-LT strains (Fig 6A). Bacteria-laden macrophages expanded to fill alveolar spaces, with no evidence of containment of the inflammatory process. In addition, multiple small lymphoid aggregates were present throughout the lung as well as foci of acute neutrophilic inflammation (Fig 6B). Besides the widespread destruction of lung parenchyma, fluid and inflammatory cells were present in remaining airways (Fig 6C). Of note, throughout the course of infection, we observed 20% (6 of 30 mice) and 12% (3 of 25 mice) mortality in Mtb-LT1 and Mtb-LT2, respectively. This can be attributed to the replacement of alveolar surfaces by the rapidly progressing inflammatory process seen in these animals. Although there were far more bacilli present in the lungs of Mtb-LT infected mice, AFB staining demonstrated that these were primarily intracellular (Fig 6D).

In addition, within the granulomatous lesion of Mtb-LT infected animals we found foamy macrophages and lipid clefts similar to those found in atheromatous plaques in patient with hypercholesterolemia (S5 Fig). These solid lipid crystals, dissolved by the solvents used in tissue processing to leave clefts, are a source of inflammation as well as mechanical injury [51]. To further confirm that the differences in the pattern of disease pathology is not confounded by examining a single lobe for histopathology, we performed another infection with Mtb-HT3 (n = 4) and Mtb-LT3 (n = 4) and analyzed multiple lung lobes from 12 week-infected animals. In line with earlier experiments, all of the Mtb-HT3 infected animals developed discrete granulomas and 2 of the 4 showed caseating granulomas (S6A Fig) in one lobe while 100% of Mtb-LT3 infected mice exhibited diffused inflammatory pathology that was present in all lung lobes (S6B Fig). Together, these data confirm that Mtb-HT and Mtb-LT infected mice develop strikingly different pathological disease.

Combined histological evaluations of 12 and 16 week-infected lungs revealed that only a proportion of Mtb-HT infected animals developed caseating granulomas (Table 2). The rest of the mice exhibited discrete granulomas with the potential to progress to caseous necrotic granulomas. Of significance, none of the Mtb-HT infected animals at either 12 or 16 week of infection exhibited diffused inflammation. In contrast, all of the Mtb-LT1 and Mtb-LT2 infected animals developed diffused inflammation with no evidence of caseating or discrete granulomas in any of the mice. Notably, despite having lung bacterial numbers similar to Mtb-HT infected mice, 8 of 9 mice of the Mtb-LT3 infected animals developed diffused inflammation and none of these infected mice had caseating granulomas (Table 2). If we consider the two pathological outcomes, caseating granulomas and diffused inflammation between Mtb-HT1-3 and Mtb-LT1-3 as a primary endpoint, a two-sided Fisher’s exact test yields a p value of p<0.0001. This suggests that the 3 Mtb-HT and 3 Mtb-LT strains have significantly different pathological outcomes.

**Table 2:** Type of granuloma development in Mtb-HT and Mtb-LT infected mice between 12–16 weeks following infection.

Number of animals with	Mtb-HT1 n = 9	Mtb-HT2 n = 9	Mtb-HT3 n = 10	Mtb-LT1 n = 10	Mtb-LT2 n = 10	Mtb-LT3 n = 9
Caseating granulomas	5	3	5	0	0	0
Diffused inflammation	0	0	0	10	10	8
Compact granulomas	4	6	5	0	0	1

In parallel, we also evaluated the lung pathological response in C57BL/6 and BALB/c animals that were infected with Mtb-HT1 and Mtb-LT1 strains. Quantification of granulomatous inflammation in both mouse genotypes established that at 12-weeks post infection, animals infected with Mtb-LT1 strain had significantly higher lung area involvement as compared with Mtb-HT1 infected animals (S3B Fig). Between the genotypes, BALB/c mice infected with Mtb-LT1 had significantly more lung area involvement at this time point (S3B Fig).

### Enhanced lipid droplet formation in macrophages infected with Mtb-LT strains

**Fig 7:**
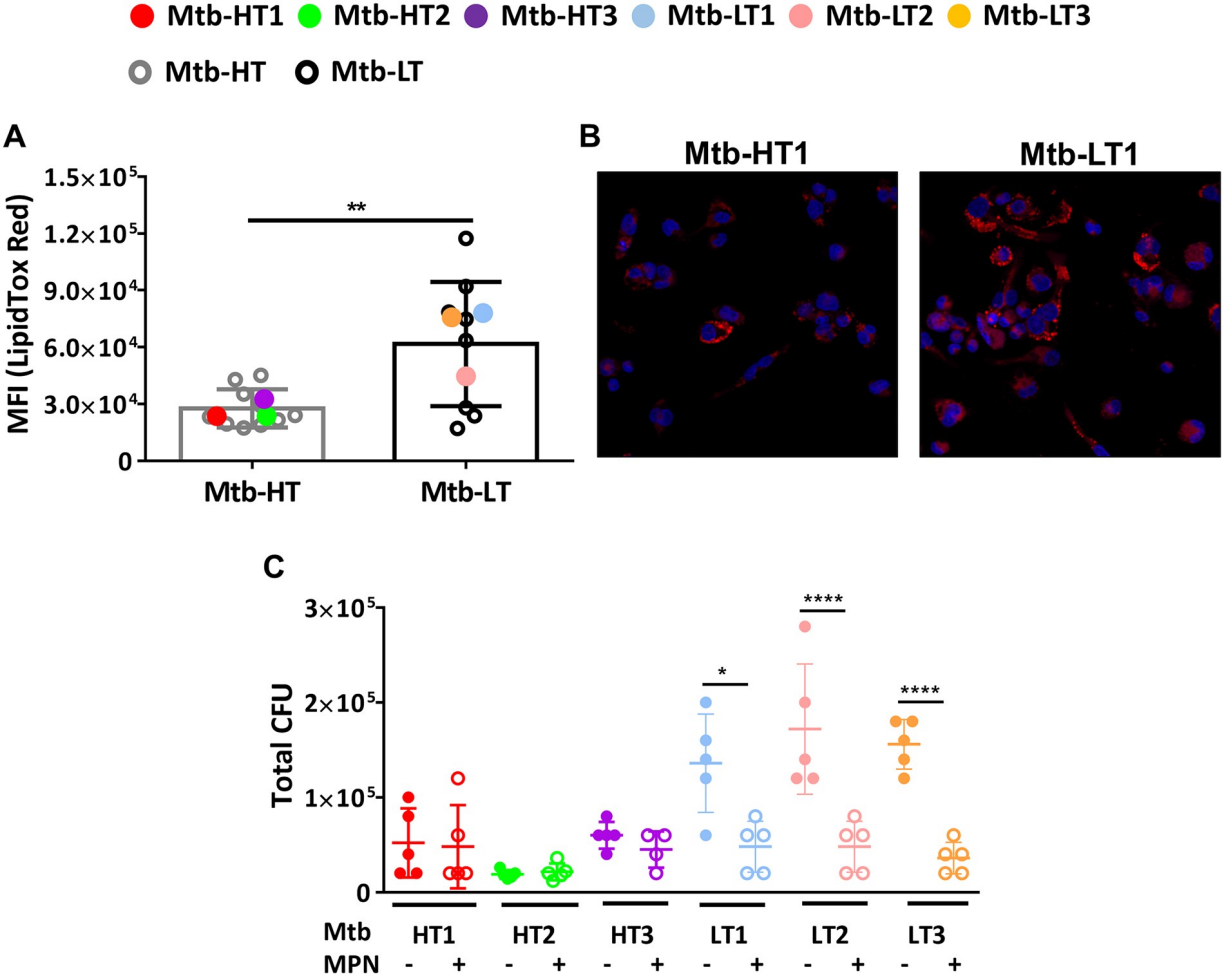
Increased growth of Mtb-LT strains in macrophages is linked to increased lipid droplet accumulation. MH-S cells were infected in triplicate with Mtb-HT and Mtb-LT strains at an MOI of 10. A total of 10 Mtb-HT and 10 Mtb-LT strains were analyzed, and it included the three Mtb-HT and three Mtb-LT strains described previously. Two days post-infection, cells were fixed in 4% paraformaldehyde, following which they were incubated with HCS LipidTOX Red dye. Mean Fluorescence intensity (MFI) is represented for LipidTOX Red. The average data for each strain is presented as a dot and the cumulative data as interquartile range with median. The data are representative of 1 of 2 individual experiments. Unpaired t-test was used to calculate the significant differences between cells infected with Mtb-HT and Mtb-LT strains (**p<0.01) (A). MH-S cells were grown on coverslips, infected with either Mtb-HT1 or Mtb-LT1. 48 hours later cells were processed for confocal imaging. The data is a representative confocal imaging showing cell nuclei stained with DAPI (blue) and presence of neutral lipids detected through HCS LipidTOX Red dye (B). BMDMs were differentiated for 7 days in D-10 containing 20% L-cell. Cells were infected using 3 MOI of the indicated strains (5 replicate wells for each condition). Data presented are day 7 CFU with and without MPN (final concentration of 100nM) (C) and are representative of one of three individual experiments. Data is represented as mean +/- SEM and significant differences in intracellular bacterial growth were calculated using one way ANOVA (*p <0.05 and ****p <0.0001).

Central to the intracellular growth of Mtb is its ability to induce foamy macrophage by triggering the accumulation of lipid droplets, which are composed of triglycerides and cholesteryl esters [52, 53]. Lipid droplets thus provide a nutrient source to intracellular Mtb and promote successful replication of the pathogen in the host [54, 55]. We argued that the difference in the growth of Mtb-LT and Mtb-HT *in vivo* in mice is due to their differential ability to induce lipid droplet formation. MH-S cells, a mouse lung alveolar macrophage cell line, were infected individually *in vitro* at an MOI of 10 with the 3 Mtb-HT and 3 Mtb-LT strains studied so far and with an additional 7 each of Mtb-HT and Mtb-LT strains. Based on flow cytometric analysis, cells infected with Mtb-LT strains had a strong signal for the LipidTOX dye (a reagent that stains neutral lipid droplets) and overall significantly high MFI as compared to cells infected with Mtb-HT strains (Fig 7A). Representative images from confocal microscopy supported the flow cytometry observations (Fig 7B). These data suggest that induction of lipid droplets may be differentially regulated by Mtb-HT and Mtb-LT strains.

Mepenzolate bromide (MPN) was shown previously to reduce lipid droplet formation in Mtb-infected macrophages by targeting the anti-lipolytic G protein-coupled receptor GPR109A which resulted in enhanced TAG turnover [52]. Consistent with lipid droplets serving as a nutrient source for Mtb, growth of Mtb *in vitro* in macrophages was reduced in the presence of MPN [52]. Therefore, to determine if there was a link between lipid droplet formation and increased Mtb-LT growth, bone marrow macrophages were infected with the three Mtb-HT and 3 Mtb-LT strains, with or without MPN, and intracellular bacterial growth was determined at day 7 following infection. Firstly, we found that the intracellular growth of Mtb-LT strains was significantly higher than Mtb-HT strains. Furthermore, MPN treatment selectively decreased intracellular bacterial growth of only Mtb-LT strains (Fig 7B). However, bacterial growth in liquid culture of all three Mtb-HT and Mtb-LT1 and Mtb-LT2 strains was significantly inhibited by MPN. Mtb-LT3 had a slow growth rate in vitro and addition of MPN did not further reduce growth. (S7A Fig). Although, the target of MPN in Mtb remains unclear, nonetheless, these data indicate that *in vitro* growth characteristics of Mtb-HT and Mtb-LT strains are not predictive of its intracellular growth pattern in macrophages, the latter we propose being more relevant to *in vivo* infections and further that lipid droplet formation is a factor in the enhanced intracellular growth of the Mtb-LT strains. Signaling via GPR109A activates inflammatory [56] and anti-inflammatory pathways [57]. We, therefore, tested whether the level of TNF was altered in MPN-treated macrophages and found no significant difference in the production of the cytokine between macrophages with or without MPN treatment (S7B Fig). Overall, these data indicate that Mtb-LT strains induce significantly higher lipid droplets to promote their intracellular growth.

## Discussion

In this study, Mtb clinical strains were carefully characterized on the basis of epidemiological data into high and low transmission groups and studied to gain insight into the pathogenic mechanisms leading to their transmission phenotype. The experimental results demonstrate that Mtb-HT and Mtb-LT strains exhibit differential bacterial growth and lung pathology in genotypically similar hosts. Our results also demonstrate early modulation of lipid biogenesis by Mtb-LT strains which could be the likely mechanism dictating the differential outcome of infection between Mtb-HT and Mtb-LT-infected animals. Data arising from this study also advance the C3HeB/FeJ mouse model as a tractable system to identify bacterial determinants that interact with the host immune response to cause the type of pathological disease that enables different extent of bacterial transmission.

Previous studies have shown that C3HeB/FeJ mice infected with Mtb Erdman or H37Rv develop caseating granulomas and exhibit granulocytic tuberculous pneumonia referred to as Type I and Type II lesions, respectively [48]. Interestingly though, the same Mtb strain induced both lesion types. The novel observation in this study is that the granulomas evolved to caseation characterized by fibrous encapsulation with central liquefaction necrosis similar to Type 1 lesions only in Mtb-HT infected animals. Although, caseating granulomas did not develop in all of the Mtb-HT infected mice, nonetheless, none of them presented with diffused inflammatory pathology. In contrast, all of the Mtb-LT-infected mice rapidly developed diffused inflammatory pathology, but none presented with caseating granulomas. The bacterial burden in mice infected with Mtb-LT3, although similar to Mtb-HT at later time points of infection, was nonetheless significantly higher than the three Mtb-HT strains at week 2 of infection. Of note, the Mtb-LT3 exhibited a pathological response that was very similar to the two other Mtb-LT strains. This suggests that the early events leading to differences in bacterial growth in Mtb-HT and Mtb-LT-infected animals may dictate the divergence in pathological response. The genetic differences in Mtb-HT and Mtb-LT strain and the ensuing specific interactions with the host may be driving the development of predominantly one or the other lesion types in the infected hosts. Consistent with this idea, a recent study in guinea pigs also found that strains that do not transmit disease caused more inflammatory pathology compared to high transmission strains [58].

The difference in bacterial replication and inflammation between the Mtb-HT and Mtb-LT strains in the C3HeB/FeJ mice was also recapitulated in two other mouse genotypes, indicating that bacterial factors likely contribute to the differential pathological outcome of Mtb-HT and Mtb-LT infections. A comparative genomics study of a panel of 19 clinically and epidemiologically characterized isolates of Mtb found that those with greater genome deletions caused significantly less pulmonary cavitation suggesting that pathogenicity is linked to bacterial factors [59]. That bacterial genotype contributes to the transmission phenotype of the host is also indicated by the finding that a large sequence polymorphism in a gene encoding molybdopterin oxyreductase was associated with clustering [60] and an Mtb strain associated with a large outbreak in the UK harbored an insertion in an intergeneic region Rv2815-2816c [61]. Whole genome sequencing and evolutionary convergence analysis of 100 strains either least or most likely to be transmitted revealed that five Mtb genes were shared by the transmissible strains. Importantly, the Mtb strains with mutations variably affected monocyte and lymphocyte cytokine production and neutrophil generation of reactive oxygen species [62]. Together, these findings indicate that differences in bacterial factors could regulate the extent of TB transmission occurring in a host. However, future studies should determine if host genetics synergize with bacterial factors to enhance transmission. Diversity Outbred (DO) [63, 64] and Collaborative Cross (CC) mice are highly heterogeneous populations that provide a tractable experimental system to model the genetic diversity of the human outbred population. The outcome of Mtb infection in DO [65] and CC [66] mice was highly varied, ranging from resistance to high susceptibility, and was associated with a diverse range of pathological responses. Of note, the susceptible mice exhibited necrotizing tuberculous pneumonia, similar to the pathological response of C3HeB/FeJ mice infected with Mtb-LT. In the DO and CC mice, the same strain induced different pathological responses whereas in our study strain variation contributed to the different pathological disease outcome. An integrated approach that combines Mtb-HT and Mtb-LT infections in heterogenous DO and CC mice will provide a powerful means to investigate whether transmissibility is the combinatorial effect of host and strain genetic diversity.

Mtb-HT and Mtb-LT strains induce distinct immunopathological responses in the susceptible host and thereby create an environmental context that we posit is differentially permissive to transmission between the susceptible host and an exposed individual. The finding that there are higher levels of Mtb-LT organisms in granulocytic pneumonitis in mice, if directly relevant to humans would not necessarily equate to their greater abundance in infectious aerosols. Rather, it is bacterial replication in area of caseation necrosis in granulomas that is associated with cavity formation and also perhaps differential survival in aerosols that lead to increased transmission. The C3HeB/FeJ mice are disease susceptible, however, a limitation of the model is that not all Mtb-HT infected animals developed caseating necrotic granulomas, and furthermore, none of the mice developed cavitary disease, a key pathological feature of human TB. A reason for why the Mtb-HT infected mice did not develop cavitary disease may be because mice lack a functional ortholog of human MMP1 that causes matrix destruction in TB [67]. However, pulmonary cavitation in C3HeB/FeJ mice was detected after aerosol infection with Mtb by serial computed tomography (CT) imaging [68]. Together, these findings suggest that further refinement of the model such as crossing MMP-1 transgenic mice with C3HeB/FeJ or the B6.C3H-*sst1* mice [69] may provide a superior mouse model to study TB pathogenesis and transmission.

The formation of lipid-filled foamy macrophages is a hallmark of Mtb infection [53]. In mycobacteria infected cells, lipid droplets are found in close apposition to the phagosome [70]. Lipid droplet and phagosome interaction leads to engulfment of mycobacteria into the lipid droplet, providing the microbe unrestricted access to host lipids [70, 71]. Accumulation of triacylglycerol-rich lipid bodies has been shown to aid mycobacterial survival and host neutral lipids can further be stored within the bacilli as intracytoplasmic lipid inclusions, thus acting as an energy source and enhancing bacterial growth in granulomatous lesions [54, 55]. Thus lipid droplets play a prominent role in sustaining successful Mtb survival and replication in the host. What bacterial factors from Mtb-LT strains activate the GPR109A to induce the rapid accumulation of lipid droplets in macrophages awaits clarification, nonetheless, the findings from this study show that Mtb-LT exploits this host signaling pathway to its advantage. A recent study argues that lipid droplet formation is not a bacterially driven process during infection with the laboratory derived Mtb Erdman, but instead is dependent on IFNγ, and is not a source of host lipids for the pathogen [72]. Our findings that Mtb-HT induce significantly less lipid droplets and that their intracellular growth is not affected by MPN, suggests that, like Mtb Erdman, lipid droplets may not be a source of lipids for Mtb-HT. However, lipid droplet formation by Mtb-LT and their decreased growth in the presence of MPN indicates that lipid droplets specifically contribute to the enhanced intracellular growth of Mtb-LT strains. The data we present show that Mtb-LT strains engage the GPR109A pathway that is known to suppress TAG turnover to enhance lipid droplet accumulation. However, Mtb can also induce host cell lipid synthesis [73] and use triacylglycerol to accumulate lipid droplets [74]. Future investigations should explore these possibilities. Additionally, whether other factors, such as ability to survive aerosol stress also contributes to the overall transmissibility of an Mtb strain needs further inquiry.

From the standpoint of bacterial dynamics, how Mtb-LT strains survive and propogate in the community remains an interesting question. In fact, the low fitness for transmissibility of Mtb-LT strains may portend a future decline in prevalence in the community unless the strains show increased propensity to progress to disease. It is possible as well that Mtb-LT transmission is enhanced in hosts with co-morbidities, such as HIV infection, diabetes or malnutrition and in them rapid transmission to disease maintains the strains in the community.

The overall findings from this study are that Mtb-HT and Mtb-LT strains belonging to the same lineage differ in their interaction with the host immune system leading to different trajectories in bacterial growth and in the development of disease pathology. The divergence in disease pathology is likely the underlying cause of differences in infectiousness of the source case. However, since the current findings are based on a small sample size, larger confirmatory studies are required for broader inference that individual strains have biological properties that induce different pathological response that affects their transmission potential. Bearing in mind this limitation, the planned next stage of this work is to define an *in vitro* immune phenotype correlating with the *in vivo* growth and pathology to provide a high throughput screening method for validation of the current findings in a large panel of Mtb-HT and Mtb-LT strains. Furthermore, findings from ongoing studies of whole genome sequencing and metabo-lipidomic profiling of a large panel of HT and LT strains will uncover key genes and bacterial factors responsible for the dichotomy in pathogenesis. In the future, this can be translated into transmission interventions that target the bacteria.

## Methods

### Ethics statement

The household contact study from which the Mtb strains were derived was approved by the Comite de Ética em Pesquisa do Hospital Universitário Cassiano Antonio de Morais, and the Institutional Review Boards of Rutgers University Biomedical Health Sciences-Newark (RBHS) (formerly UMDNJ) and Boston University School of Medicine. Written informed consent and assent in Portuguese were obtained from all study participants as per the consent procedure approved by IRBs from all participating institutions.

All animal experiments described in this study conform to the Rutgers University Biomedical Health Sciences-Newark (RBHS) and Institutional Animal Care and Use Committee (IACUC) Guidelines as well as NIH and USDA policies on the care and use of animals in research and teaching. Efforts were taken to ensure minimal animal pain and suffering and when applicable, approved anesthesia methods were employed for the same.

### Bacterial strains

Clinical strains of Mtb were first grown on Lowenstein Jensen (LJ) growth media. Bacterial colonies picked from LJ slants were cultured in 7H9 media until mid-log phase. Cultures were then centrifuged at 400 RPM for 5 minutes, causing clumps to settle down in the bacterial pellet. The culture supernatant was collected, mixed with a final concentration of 20% glycerol, and was stored in 1 mL aliquots at -80°C. The stock titer was determined by plating 10-fold serial dilutions on Middlebrook 7H11 selective medium (Difco by BD, Franklin Lakes, NJ) and by counting the bacterial colonies 15–20 days later. The bacteria were passaged *in vitro* only twice to minimize any phenotypic/genotypic changes that might occur in the growing cultures.

### Aerosol infections and determination of bacterial burden

6–8 weeks old female C57BL/6J, BALB/c and C3HeB/FeJ mice were purchased from the Jackson Laboratory (Bar Harbor, ME, USA). Bacterial stocks were generated as described above. Mice were exposed for 40 minutes to nebulized bacteria at a density optimized to deliver a standard low dose of around 50–120 CFU (unless otherwise indicated) using Glass-Col Full Body Inhalation Exposure. For all infections, the actual infection dose was determined by plating total lung homogenates from a minimum of 3 mice on Middlebrook 7H11 plates at 24 hours after aerosol exposure. Lungs, spleens, mediastinal lymph nodes and livers were harvested and homogenized at indicated time points post-infection. Total CFU per organ was determined by plating 10-fold serial dilutions on Middlebrook 7H11 plates, which were counted after 28–35 days of incubation at 37°C.

### Histological assessment

Post-mortem, lungs of Mtb-infected mice were perfused with sterile PBS and fixed in 4% paraformaldehyde for seven days, followed by paraffin embedding. For histopathological analysis, 5- to 7-μm sections were cut and stained using a standard H&E protocol. Leica SCN400 F whole-slide scanner (Experimental Pathology Research Lab, NYU Langone Health) was used for scanning histological sections and images were analyzed using Aperio ImageScope. Stereoscopic images were obtained using Act-1 software from Nikon. For quantitation of granulomatous inflammation in the lung section, Image-Pro Discovery Software was used to create a grid overlay onto each photomicrographs of H&E stained lung section and numbers of points hitting areas of granulomatous infiltration were counted. Masson’s trichrome staining [75] was carried out by NJMS Histology core. For visualization of acid-fast bacilli (AFB), tissue sections were stained using the Ziehl-Neelsen method.

For immunohistochemical detection of Ly6G^+^ cells, tissue samples were de-paraffinized with xylene and rehydrated with ethanol gradations and water. The samples were subjected to heat-induced antigen retrieval by microwave warming using 10 mM citrate buffer (pH 6.0). Endogenous peroxidase activity was blocked using 0.3% hydrogen peroxide and then subsequently blocked with 1× PowerBlock (BioGenex). PBS containing 0.05% Tween-20 was used to wash tissues in between steps. For each sample, serial sections were incubated with the primary anti-mouse Ly6G antibody (clone 1A8; Biolegend) at a 1∶250 dilution or with isotype control (Rat IgG2a, κ; BioLegend) at the same concentration. Sections were subsequently incubated with biotinylated secondary antibody (1∶100 Vector Laboratories). Streptavidin horseradish peroxidase (BioGenex) was used to label the secondary antibody for immunodetection by DAB chromogen (BioGenex). After counterstaining with Mayer’s hematoxylin (BioGenex), the samples were dehydrated with ethanol gradations, dipped in xylene, and mounted using Cytoseal-60 (ThermoFisher). Histopathological evaluations were performed with blinding to the identity of the strain.

### Flow cytometry

Single-cell lung suspensions were prepared and cell viability was determined using Trypan-blue exclusion method. For surface staining, approximately 1 million cells were washed and resuspended in FACS buffer (PBS + 2% fetal calf serum (FCS) and 0.09% sodium azide) containing a cocktail with the appropriate concentrations of specific fluorochrome-conjugated monoclonal antibodies. Isotype controls were included for each. Cells were first incubated with LIVE/DEAD Fixable Aqua Dead Cell stain. Directly conjugated fluorochrome labeled antibodies were used for the following cell-surface markers: anti-mouse CD4-V450 (clone RM4-5; BD Horizon), anti-mouse CD8-AF488 (clone 53–6.7; BD Pharmingen), anti-mouse B220-PECF594 (clone RA3-6B2; BD Pharmingen), anti-mouse CD11b-PE (clone M1/70; BD Pharmingen), anti-mouse Ly6G-PECy7 (clone 1A8; BD Pharmingen), anti-mouse CD11c-AF700 (clone HL3; BD Pharmingen) and anti-mouse Ly6C-PerCPCy5.5 (clone HK1.4; eBiosciences). Following surface staining, samples were fixed in 4% paraformaldehyde for 30 minutes and then acquired on a LSRII flow cytometer (BD Biosciences). Analysis was performed using FlowJo software (Tree Star, Inc.). Gating was based on fluorescence minus one (FMO) controls.

For detection of lipid bodies, infected cells were fixed with Cytofix/Cytoperm solution (BD) for 20 minutes. Then, cells were washed twice with Perm/Wash buffer (BD) and cells were then resuspended in PBS solution of HCS LipidTOX Deep Red Neutral Lipid stain (ThermoFisher Scientific). Following this incubation step, cells were washed twice with PBS and resuspended in PBS for flow cytometric analysis.

### Confocal microscopy

Adherent MH-S cells were grown on coverslips (Fisherbrand) placed in 6-well plates (Corning). Following Mtb infection, cells were fixed in 4% formaldehyde, washed and then stained with PBS solution of HCS LipidTOX Deep Red Neutral Lipid stain (ThermoFisher Scientific), followed by 300 nM DAPI nuclear stain solution (ThermoFisher Scientific). Coverslips were then washed three times with PBS and then mounted on Super frost/Plus microscope slides using Molecular Probes Slowfade Light antifade medium. Nikon A1RS confocal microscope was used to acquire images and quantification of signal intensity was performed using ImageJ software and Nikon imaging software, Nikon Elements 4.5.

### *In vitro* quantification of intracellular mycobacterial growth

BMDMs were prepared as described previously [76]. On day 7, BMDMs were plated in 96 well plate (Corning) at a cell density of 0.08 x 10^6^ cells/well in 200μL of D10 media [antibiotic free DMEM media (Mediatech, Inc.) containing 10% defined FBS (HyClone Laboratories, Logan, UT)] and supplemented with 2% conditioned medium from L-cells. Cells were infected in replicates of 5 with 3 MOI of three Mtb-HT and three Mtb-LT strains for 4 hours. Wells were then washed 4 times with PBS + 1% BCS and cells were untreated or treated with 100nM of MPN. Infected cells were maintained in D-10 media supplemented with 2% conditioned medium from L-cells. At day 7, post-infection cells were washed with serum-containing PBS and then lysed with sterile water. Total CFU was determined by plating 10-fold serial dilutions on Middlebrook 7H11 plates, which were counted after 21 days of incubation at 37 °C. 48-hour culture supernatants were harvested for measuring TNF levels.

### Enzyme linked immunosorbent assay (ELISA)

Lung lysates from Mtb infected mice were treated with 2X protease inhibitor (ThermoFisher Scientific) at the time of collection and supernatants from infected BMDMs were sterilized using 0.22μm. Ultrafree-MC centrifugal filter (EMD Millipore). ELISA Ready-Set-Go kit was used or IL-17 (eBioscience). For all ELISA, colorimetric analyses were used to calculate protein concentration levels (Molecular Devices, Softmax Pro). For TNF, IL-1β, IL-6 and KC, a multi-analyte detection system that incorporates electro-chemiluminescence based readout was used (MesoScale Discovery, Rockville, MD, USA). Pre-coated 10-spot MULTI-SPOT plates with capture antibodies were purchased (catalog # K15048D). The assays are based on the principle of electrochemiluminescence (ECL) sandwich ELISA. The calculations to establish calibration curves and determine analyte concentrations were carried out using the MSD DISCOVERY WORKBENCH analysis software.

## Statistics

All statistical analyses were performed using Graph Pad Prism software. For analysis of two groups, the unpaired t-test was used. For greater than two groups, One- or Two- way ANOVA with Bonferroni’s correction was used. In all cases, p value <0.05 was considered to be statistically significant.

## Supporting information

S1 Fig Increased bacterial dissemination in C3HeB/FeJ mice infected with Mtb-LT strains. C3HeB/FeJ animals were infected with a low dose inoculum of Mtb-HT1, Mtb-HT2, Mtb-HT3, Mtb-LT1, Mtb-LT2, and Mtb-LT3 strains. At 4 and 12 weeks following aerosol infection, serial dilutions of spleen and liver homogenates were plated on 7H11 agar plates and the bacterial load was determined between 28–35 days of incubation at 37°C. ND = not detectable.
(PDF)

S2 Fig Bacterial burden in CeHeB/FeJ animals infected with Mtb-HT1 and Mtb-LT1. C3HeB/FeJ animals were infected with low dose of Mtb and lungs, mediastinal lymph nodes and spleens were plated at 12-weeks post infection. Two-way ANOVA showed significant difference between animals infected with Mtb-HT and Mtb-LT strains Each group includes 4–5 mice per time point and data is represented as mean +/- SEM and * = p<0.05.
(PDF)

S3 Fig Higher bacterial burden and more granulomatous lesions in the lungs of Mtb-LT1 infected C57BL/6J and BALB/c mice. C57BL/6 and BALB/c mice were aerosol infected with a low dose of Mtb-HT1 and Mtb-LT1 strains. CFU/mouse was enumerated by plating lung homogenates at indicated time points following infection (A). Data are presented as mean +/- SEM; **p<0.01, *** p<0.001 and ****p<0.0001. Formalin-fixed, paraffin-embedded lung tissue was obtained from mice at 12 weeks following infection with Mtb-HT1 and Mtb-LT1, and sections were stained using a standard H&E protocol. Comparison of lung area under granulomatous inflammation in Mtb-HT1 and Mtb-LT1 infected C57BL/6 and BALB/c mice, shows significantly higher involvement of lung tissue post Mtb-LT1 infections (B). This quantification was done using ImagePro discovery software. Each group includes 5 mice and data is represented as interquartile range with median (***p<0.005 and ^##^p<0.05).
(PDF)

S4 Fig Increased inflammatory response in Mtb-LT infected mice. Lung lysates from four week-infected C3HeB/FeJ mice were obtained after homogenizing lung tissue in 1ml PBS and 2X protease inhibitor (ThermoFisher). Levels of different immune mediators were evaluated in filtered cell-free lysates using singleplex ELISA and/or multiplex MesoScale Discovery (MSD) platform. Data are from five mice and presented as mean +/- SEM. For each cytokine, data from the three Mtb-HT infections were combined and compared to combined data from the three Mtb-LT infections using unpaired t-test. TNF: p <0.01; IL-1β: p <0.01; IL-6: p <0.01; IL-17: p <0.01; KC: p <0.001.
(PDF)

S5 Fig Presence of cholesterol crystals in lung lesions of Mtb-LT infected mice. Formalin-fixed, paraffin-embedded lung tissue was obtained from mice at 12 weeks following infection with Mtb-LT1 and sections were stained using a standard H&E protocol. Arrows point to the presence of cholesterol crystals.
(PDF)

S6 Fig Lung pathology in Mtb-HT3 and Mtb-LT3 infected mice. Multiple lung lobes from Mtb-HT3 (A) and Mtb-LT3 (B) infected C3HeB/FeJ animals were fixed, paraffin embedded and stained using H&E protocol, at week 12 post infection. Black box = presence of fibrotic lesions; *green = discrete lesion; and *yellow = diffused inflammation.
(PDF)

S7 Fig Bacterial growth and effect of MPN. Frozen stocks of the indicated strains were used to inoculate 7H9 media containing 0.05% Tween 80. The samples were allowed to grow 3 days in liquid culture reaching mid-log phase. The growing culture was split 1:100 into flasks containing 7H9 + Tween or flasks containing 7H9 + Tween and 100nM MPN. The OD 600 from aliquots of the culture was read at the indicated time points. A paired student test was performed to calculate significant difference between the MPN treated and untreated cultures Mtb-HT1:p<0.0001; Mtb-HT2:p<0.001; Mtb-HT3:p<0.01; Mtb-LT1:p<0.001 Mtb-LT2:p<0.01; Mtb-LT3:ns (A). Supernatants of infected macrophage cultures were assayed for TNF levels by ELISA. No significant differences were found between MPN treated and untreated cultures (B).
(PDF)

S1 Table TST positivity in HHC infected with Mtb-HT and Mtb-LT strains. (PDF)
