# Supplementary figures and images for: Transmission phenotype of Mycobacterium tuberculosis strains is mechanistically linked to induction of distinct pulmonary pathology
Source: PLoS Pathog. 2019 Mar 6;15(3):e1007613. doi: 10.1371/journal.ppat.1007613 (PMC6422314; doi:10.1371/journal.ppat.1007613)

S1 Fig

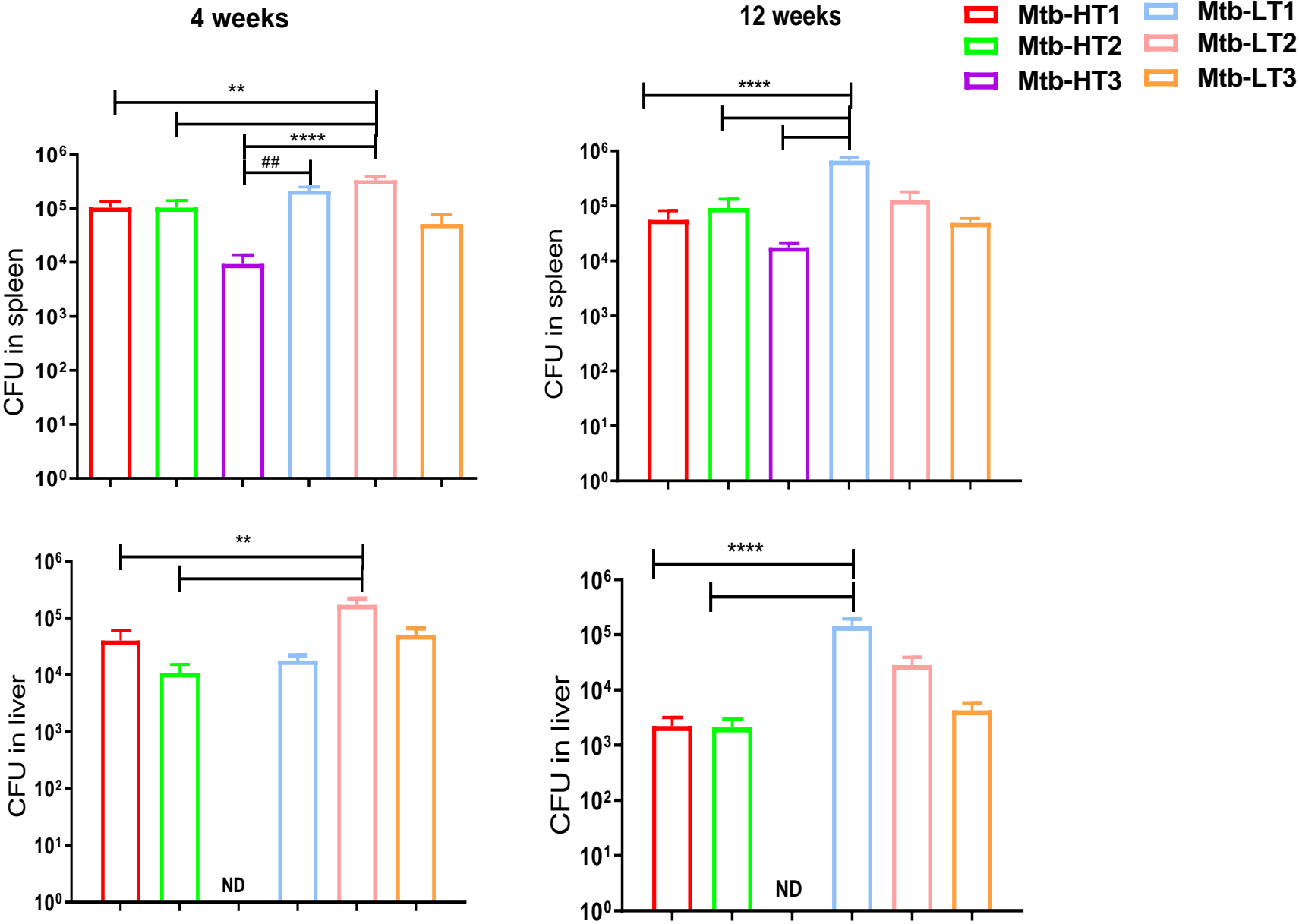

Supplement: S1 Fig — C3HeB/FeJ animals were infected with a low dose inoculum of Mtb-HT1, Mtb-HT2, Mtb-HT3, Mtb-LT1, Mtb-LT2, and Mtb-LT3 strains. At 4 and 12 weeks following aerosol infection, serial dilutions of spleen and liver homogenates were plated on 7H11 agar plates and the bacterial load was determined between 28–35 days of incubation at 37°C. ND = not detectable. (PDF) [file ppat.1007613.s001.pdf]

S2 Fig

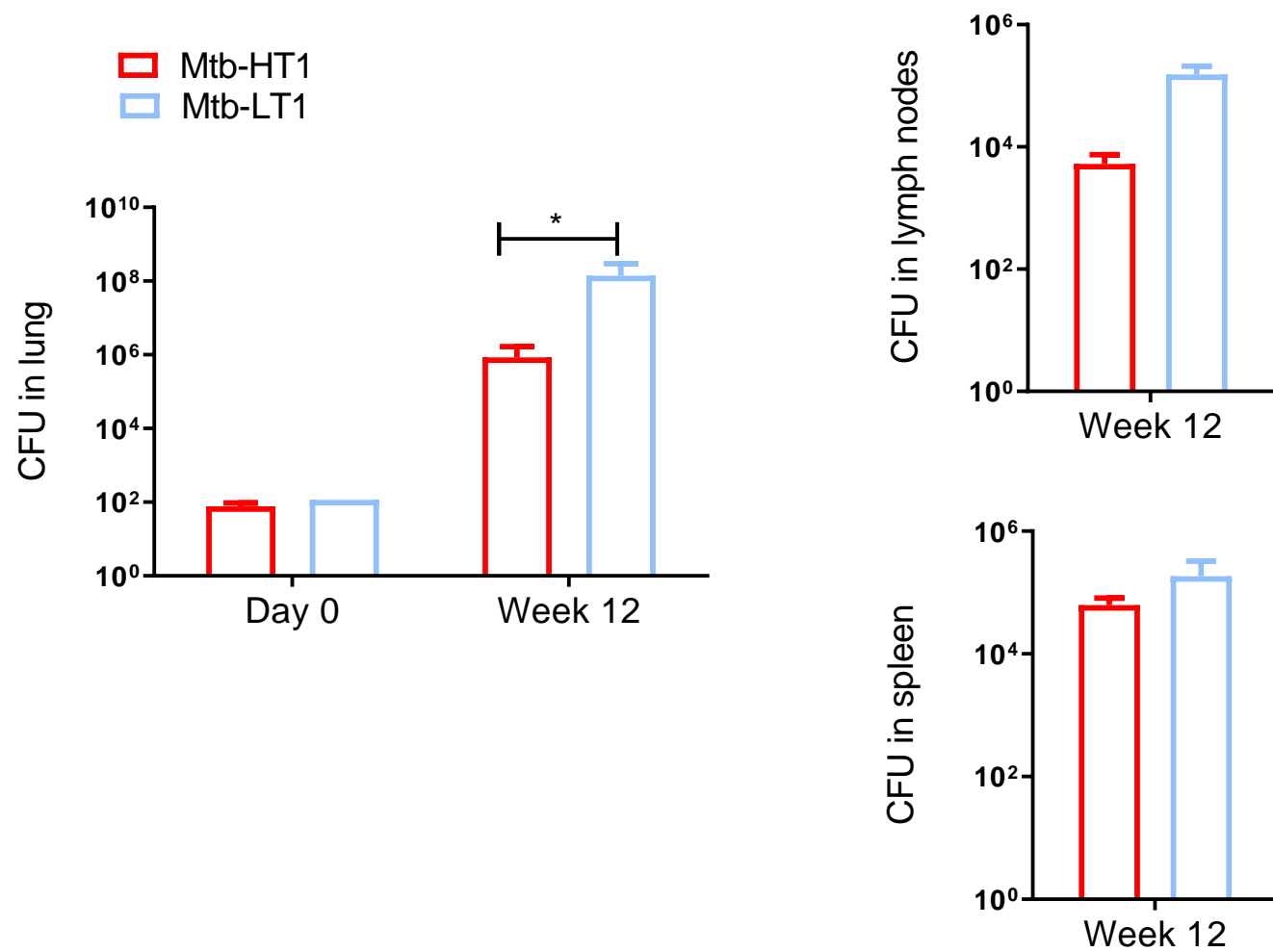

Supplement: S2 Fig — C3HeB/FeJ animals were infected with low dose of Mtb and lungs, mediastinal lymph nodes and spleens were plated at 12-weeks post infection. Two-way ANOVA showed significant difference between animals infected with Mtb-HT and Mtb-LT strains Each group includes 4–5 mice per time point and data is represented as mean +/- SEM and * = p<0.05. (PDF) [file ppat.1007613.s002.pdf]

S3 Fig

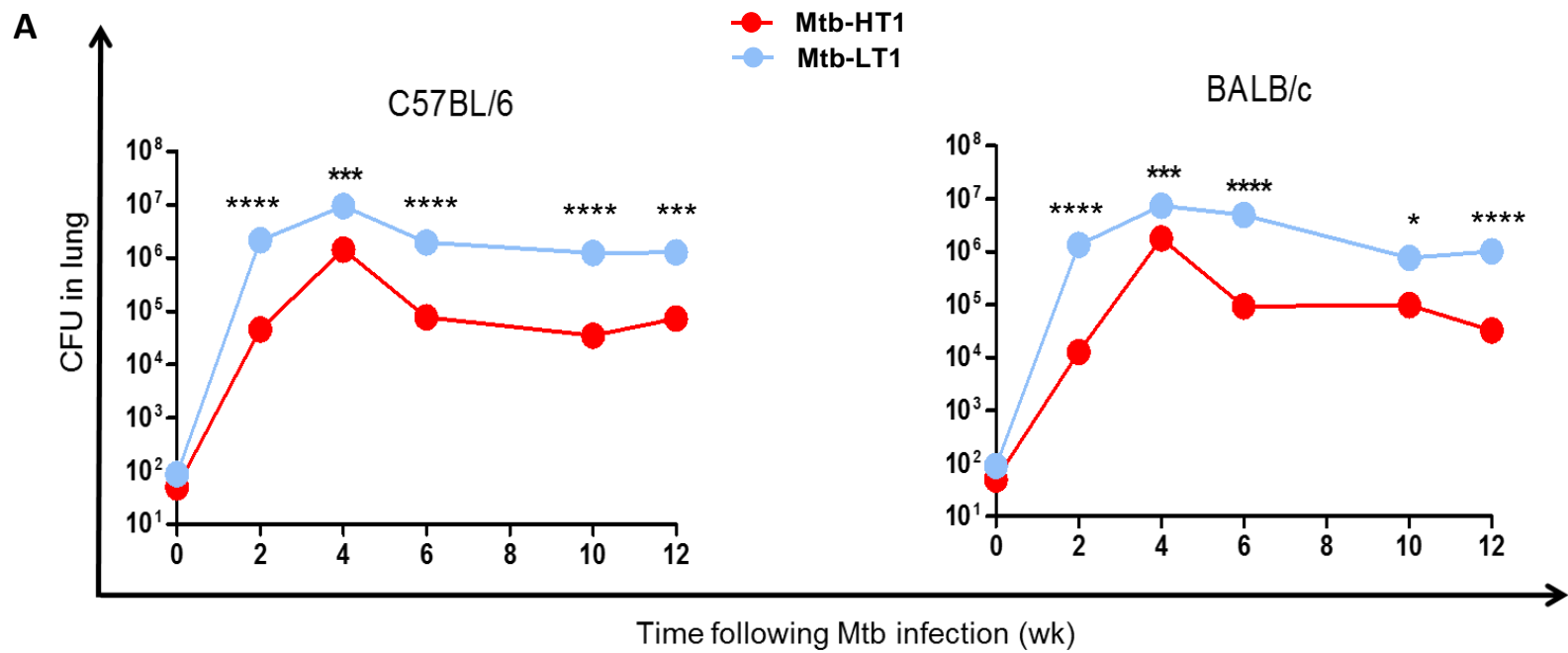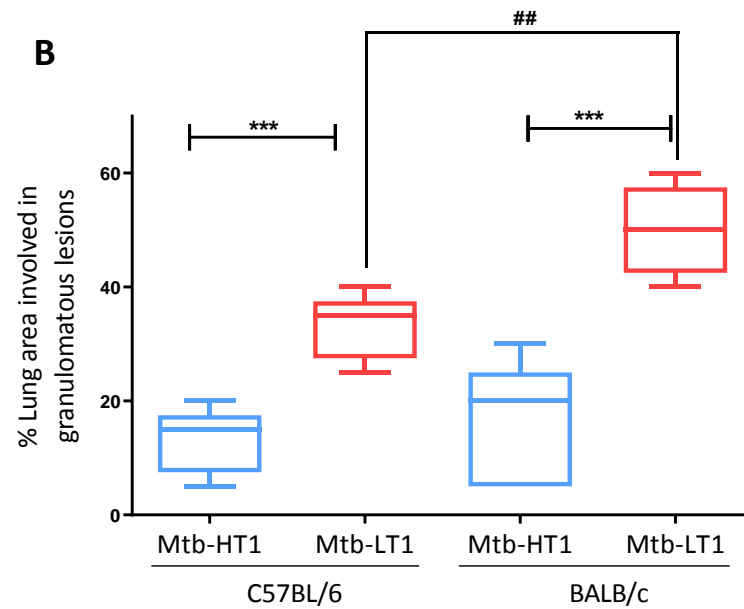

Supplement: S3 Fig — C57BL/6 and BALB/c mice were aerosol infected with a low dose of Mtb-HT1 and Mtb-LT1 strains. CFU/mouse was enumerated by plating lung homogenates at indicated time points following infection (A). Data are presented as mean +/- SEM; **p<0.01, *** p<0.001 and ****p<0.0001. Formalin-fixed, paraffin-embedded lung tissue was obtained from mice at 12 weeks following infection with Mtb-HT1 and Mtb-LT1, and sections were stained using a standard H&E protocol. Comparison of lung area under granulomatous inflammation in Mtb-HT1 and Mtb-LT1 infected C57BL/6 and BALB/c mice, shows significantly higher involvement of lung tissue post Mtb-LT1 infections (B). This quantification was done using ImagePro discovery software. Each group includes 5 mice and data is represented as interquartile range with median (***p<0.005 and ##p<0.05). (PDF) [file ppat.1007613.s003.pdf]

S4 Fig

Mtb-HT1 Mtb-HT2 Mtb-HT3 Mtb-LT1 Mtb-LT2 Mtb-LT3

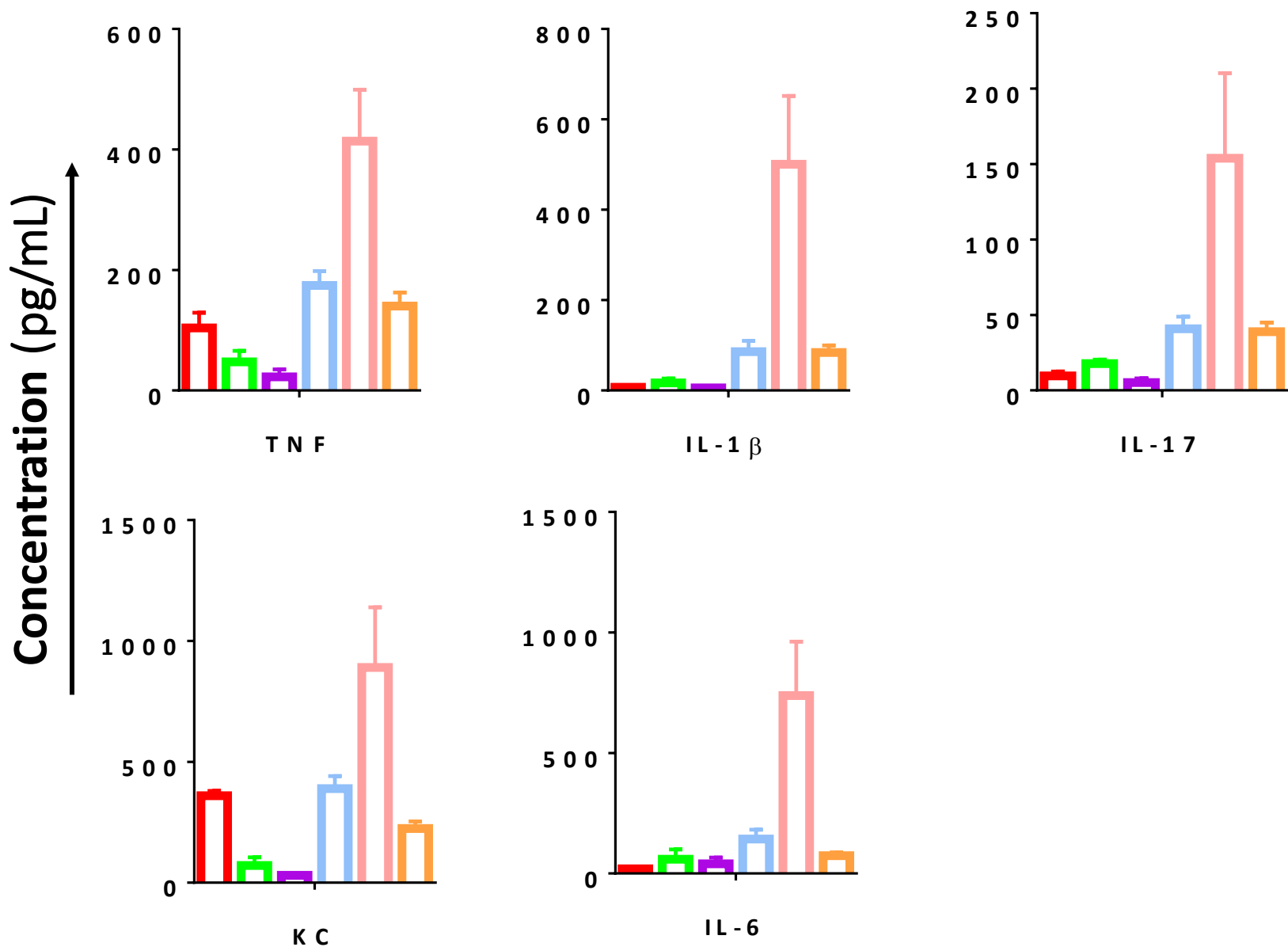

Supplement: S4 Fig — Lung lysates from four week-infected C3HeB/FeJ mice were obtained after homogenizing lung tissue in 1ml PBS and 2X protease inhibitor (ThermoFisher). Levels of different immune mediators were evaluated in filtered cell-free lysates using singleplex ELISA and/or multiplex MesoScale Discovery (MSD) platform. Data are from five mice and presented as mean +/- SEM. For each cytokine, data from the three Mtb-HT infections were combined and compared to combined data from the three Mtb-LT infections using unpaired t-test. TNF: p <0.01; IL-1β: p <0.01; IL-6: p <0.01; IL-17: p <0.01; KC: p <0.001. (PDF) [file ppat.1007613.s004.pdf]

S5 Fig

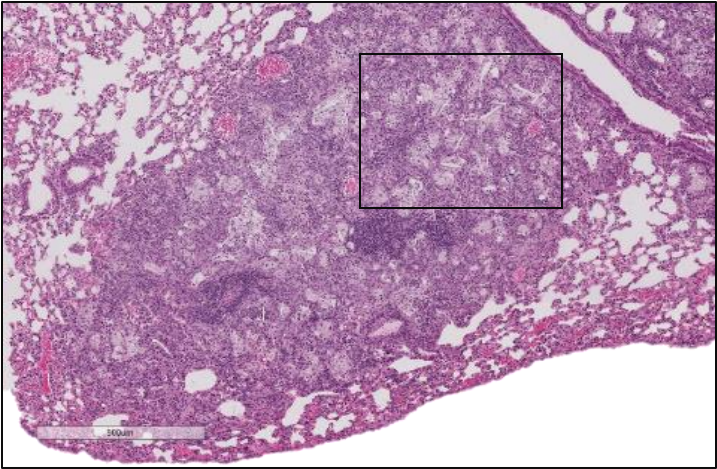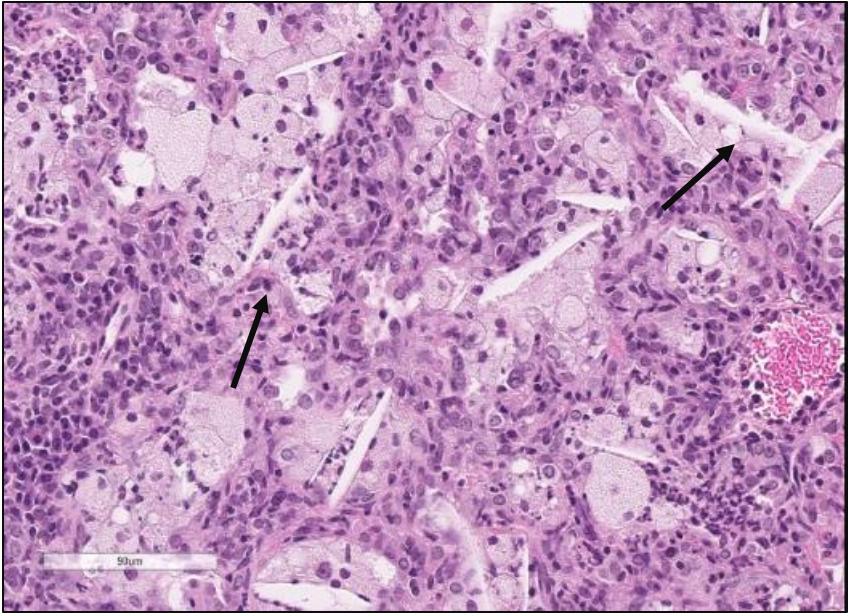

Supplement: S5 Fig — Formalin-fixed, paraffin-embedded lung tissue was obtained from mice at 12 weeks following infection with Mtb-LT1 and sections were stained using a standard H&E protocol. Arrows point to the presence of cholesterol crystals. (PDF) [file ppat.1007613.s005.pdf]

S6 Fig

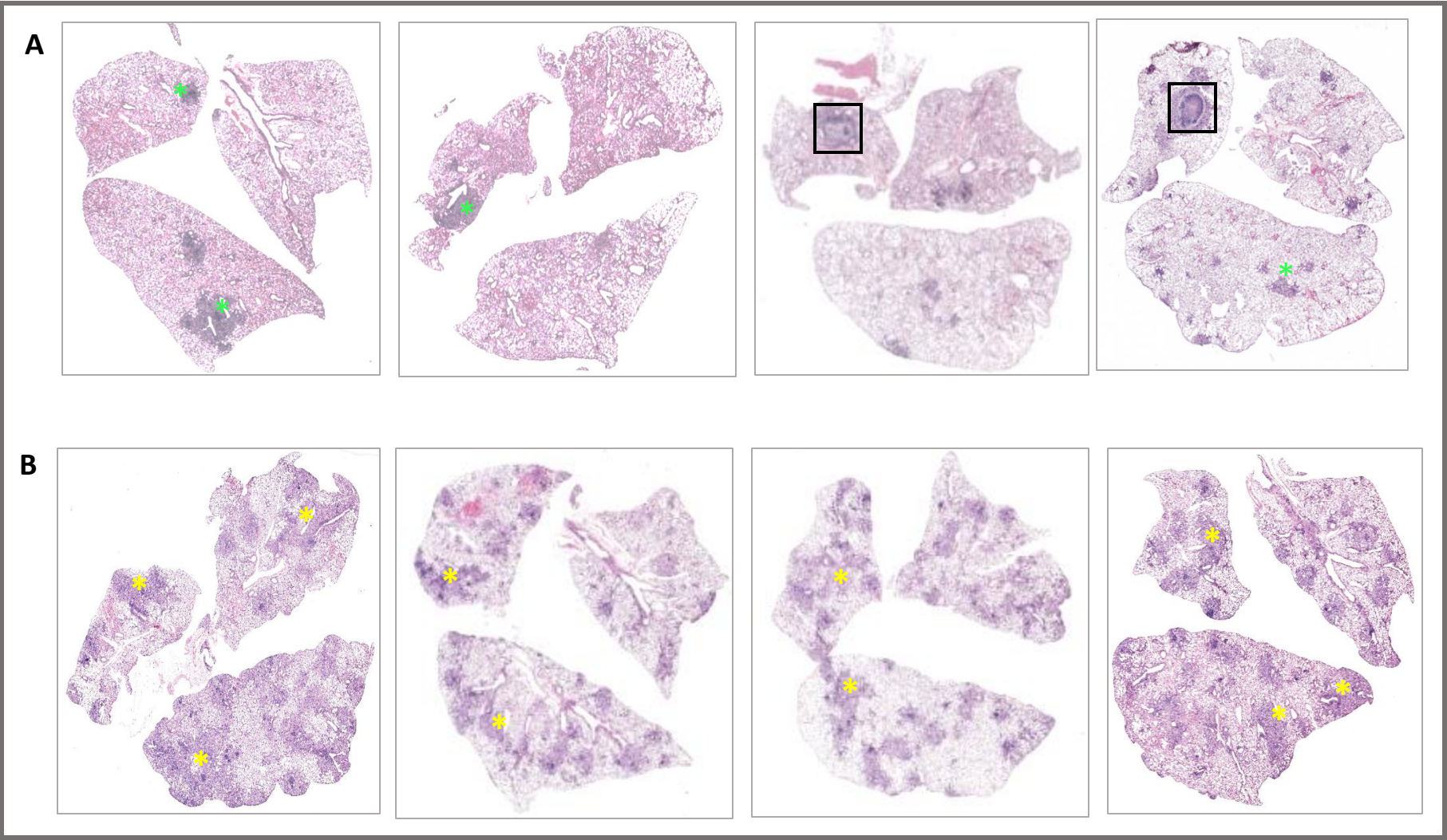

Supplement: S6 Fig — Multiple lung lobes from Mtb-HT3 (A) and Mtb-LT3 (B) infected C3HeB/FeJ animals were fixed, paraffin embedded and stained using H&E protocol, at week 12 post infection. Black box = presence of fibrotic lesions; *green = discrete lesion; and *yellow = diffused inflammation. (PDF) [file ppat.1007613.s006.pdf]

S7 Fig

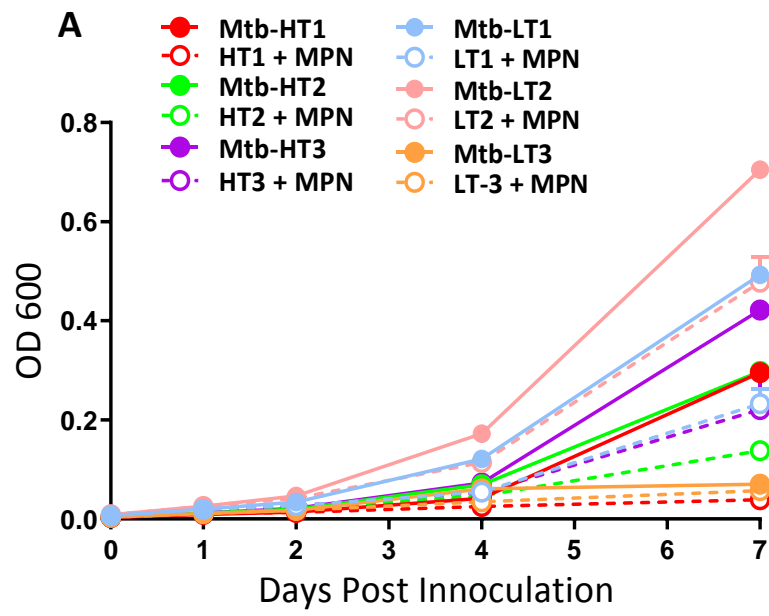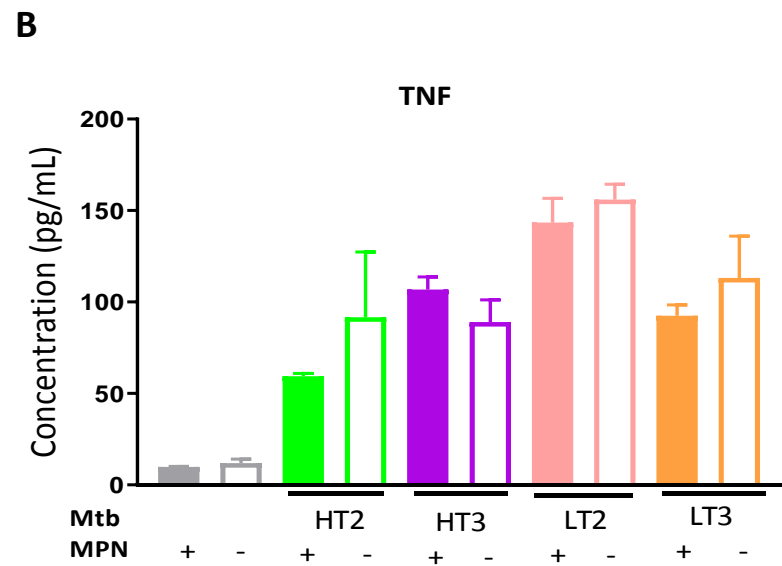

Supplement: S7 Fig — Frozen stocks of the indicated strains were used to inoculate 7H9 media containing 0.05% Tween 80. The samples were allowed to grow 3 days in liquid culture reaching mid-log phase. The growing culture was split 1:100 into flasks containing 7H9 + Tween or flasks containing 7H9 + Tween and 100nM MPN. The OD 600 from aliquots of the culture was read at the indicated time points. A paired student test was performed to calculate significant difference between the MPN treated and untreated cultures Mtb-HT1:p<0.0001; Mtb-HT2:p<0.001; Mtb-HT3:p<0.01; Mtb-LT1:p<0.001 Mtb-LT2:p<0.01; Mtb-LT3:ns (A). Supernatants of infected macrophage cultures were assayed for TNF levels by ELISA. No significant differences were found between MPN treated and untreated cultures (B). (PDF) [file ppat.1007613.s007.pdf]
