# Supplementary material for: Transmission phenotype of Mycobacterium tuberculosis strains is mechanistically linked to induction of distinct pulmonary pathology
Source: PLoS Pathog. 2019 Mar 6;15(3):e1007613. doi: 10.1371/journal.ppat.1007613 (PMC6422314; doi:10.1371/journal.ppat.1007613)
Supplement: S1 Table — (PDF) [file ppat.1007613.s008.pdf]

S1 Table. TST positivity in HHC infected with Mtb-HT and Mtb-LT strains.

**Mtb strains *in vitro* experiments**	**TST positivity in the household (%)**
Mtb-HT4	100
Mtb-HT5	100
Mtb-HT6	83
Mtb-HT7	80
Mtb-HT8	75
Mtb-HT9	80
Mtb-HT10	70
Mtb-LT4	0
Mtb-LT5	25
Mtb-LT6	0
Mtb-LT7	0
Mtb-LT8	24
Mtb-LT9	25
Mtb-LT10	13
